# Medical Student Research: An Integrated Mixed-Methods Systematic Review and Meta-Analysis

**DOI:** 10.1371/journal.pone.0127470

**Published:** 2015-06-18

**Authors:** Mohamed Amgad, Marco Man Kin Tsui, Sarah J. Liptrott, Emad Shash

**Affiliations:** 1 Faculty of Medicine, Cairo University, Cairo, Egypt; 2 Okinawa Institute of Science and Technology Graduate University, Okinawa, Japan; 3 European Institute of Oncology (IEO), Milano, Italy; 4 National Cancer Institute, Cairo University, Cairo, Egypt; Kyoto University, JAPAN

## Abstract

**Importance:**

Despite the rapidly declining number of physician-investigators, there is no consistent structure within medical education so far for involving medical students in research.

**Objective:**

To conduct an integrated mixed-methods systematic review and meta-analysis of published studies about medical students' participation in research, and to evaluate the evidence in order to guide policy decision-making regarding this issue.

**Evidence Review:**

We followed the PRISMA statement guidelines during the preparation of this review and meta-analysis. We searched various databases as well as the bibliographies of the included studies between March 2012 and September 2013. We identified all relevant quantitative and qualitative studies assessing the effect of medical student participation in research, without restrictions regarding study design or publication date. Prespecified outcome-specific quality criteria were used to judge the admission of each quantitative outcome into the meta-analysis. Initial screening of titles and abstracts resulted in the retrieval of 256 articles for full-text assessment. Eventually, 79 articles were included in our study, including eight qualitative studies. An integrated approach was used to combine quantitative and qualitative studies into a single synthesis. Once all included studies were identified, a data-driven thematic analysis was performed.

**Findings and Conclusions:**

Medical student participation in research is associated with improved short- and long- term scientific productivity, more informed career choices and improved knowledge about-, interest in- and attitudes towards research. Financial worries, gender, having a higher degree (MSc or PhD) before matriculation and perceived competitiveness of the residency of choice are among the factors that affect the engagement of medical students in research and/or their scientific productivity. Intercalated BSc degrees, mandatory graduation theses and curricular research components may help in standardizing research education during medical school.

## Introduction

The education of health professionals has seen two revolutions over the past century. The first revolution-marked by what is known as The Flexner Report in 1910- was the effective integration of basic sciences into health education. The second revolution, initiated by the Welch-Rose report in 1915, introduced the concept of problem-based learning into medical education. In 2010, a special report was published by a global commission, The Commission on Education of Health Professionals for the 21^st^ Century, aimed at updating the standards of an ideal medical curriculum. The committee strongly recommended a new medical educational model that emphasized flexibility and adaptability of traditionally rigid curricula to local and community needs [1]. Despite these educational advances, there are certain aspects of medical education that remain unstructured and largely variant between medical schools; among these is medical student participation in research. Moreover, there is an alarming decline in the number of physician-scientists in the US, which threatens the progress of translational medicine in the upcoming era [2–4].

In the U.S., outstanding students willing to enter medical school may apply for the National Institute of Health (NIH) funded Medical Scientist Training Program (MSTP) [5]. This program offers students the opportunity to get a good feel for what a physician-scientist career entails through a funded MD/PhD. The value of those MD/PhD programs is well established; a 2010 study by Brass et al, investigating the outcomes of half of all NIH-funded MD/PhD programs (24 programs in total) found that these programs were very successful at reaching their goals of training future physician-scientists. In fact, 81% of MD/PhD graduates landed academic positions and 82% of them were actively engaged in research [6]. Nevertheless, due to limited funding, MD/PhD graduates only constitute 3% of the US medical student population, highlighting the value of alternative pipelines for the creation of research-active physicians [7]. Moreover, organizational and contextual factors might make the support of costly MD/PhD programs difficult to implement in other countries.

Several other programs have also been devised to offer medical and health sciences students the chance to participate in research [8–13]. One of the common forms of medical student research engagement is Intercalated Bachelor of Science (iBSc) degrees. These are particularly common in the UK, and are characterized by research time-out periods between the basic and clinical years of medical school. Students who take intercalated degrees graduate with an extra BSc beside their medical degree. The value of such short-term research placements should not be underestimated. In fact, the benefits of undergraduate research have been discussed richly in the literature, though there were relatively fewer papers focusing primarily on medical student research [14–16]. Unlike many other degrees, a medical degree is at the interface of science and social service. It is therefore expected that the benefits of, and motivations behind, medical student participation in research are different from those of non-medical students [17].

A 2005 systematic review of the literature by Straus et al investigated the factors that influence career choice in academic medicine among residents, fellows and staff physicians [18]. Their review found a positive effect of having dual degrees or fellowships beside the medical degree, and of publishing research conducted during medical school. Further, the review highlighted the role of mentorship and desire to teach. Despite the presence of a large body of evidence investigating the impact of, and factors related to, medical student research, a systematic analysis of this evidence is missing. This makes the data seem conflicting and disorganized, and undermines the apparent overall strength of evidence.

This paper is a mixed-methods systematic review and meta-analysis of published studies investigating various aspects of medical student research, including its impact on the development of research-active physicians, difficulties faced by medical students performing research and potential solutions to overcome these difficulties. Our hope is that this work serves to complement the review by Straus et al, and helps provide a thorough overview of the evidence needed for curricular and educational policy reforms [18].

We aimed to satisfy the following objectives in this review:

**Primary Objectives:** (a) To examine the short- and long- term influence of curricular and extracurricular undergraduate medical research on the scientific productivity of medical students, measured by the number of published manuscripts, research awards or attainment of faculty rank. (b) To describe the influence of curricular and extracurricular medical student research on the career choice of medical students.

**Secondary Objectives:** (a) To explore the current forms in which medical students are engaged in research projects, as well as the prevalence of non-mandatory research exposure among medical students. (b) To identify the factors related to medical student engagement in research projects. (c) To investigate miscellaneous issues of relevance, including the pros and cons of research time-out periods (with a focus on Intercalated Bachelor of Science degrees), differences between countries with developing and developed economies and gender equality in medical student research engagement, perceptions and productivity.

Developing economies were identified according to the International Monetary Fund's World Economic Outlook Report [19]. We counted as a "medical student" anyone who is enrolled in the core medical school program, regardless of program duration, and whose graduation would guarantee the degree Bachelor of Medicine, Bachelor of Surgery (MBBS) or its equivalent (MD, in the US, for example). It should be noted that in the US model of medical education, admission into medical school is on a graduate-entry basis by default, and the first medical degree earned is called the "MD". In the non-graduate entry model, on the on the other hand, the term "MD" is reserved for higher research degrees (postgraduate degrees) in clinical medical and surgical disciplines. Graduate-entry medical students were included, but not MD/PhD students, residents or postgraduate students. The reasons behind excluding studies focusing on MD/PhD students is that this sub-population is considered to be different from the general student population, especially that their enrollment in the medical program was–by definition- meant to prepare them for physician-scientist careers. It may be argued that graduate-entry medical students who had a higher degree (MSc or PhD) at the time of matriculation also constitute a separate sub-population. Hence, we addressed any reported differences between these sub-populations in our results. "Medical student research" was defined as any activity performed by medical students that is driven by inquiry or hypothesis and that legitimately incorporates basic principles of the scientific method. This includes original research, review articles, case reports etc.

## Methods

We followed the PRISMA (Preferred Reporting Items for Systematic Reviews and Meta-Analysis) statement guidelines in this systematic review and meta-analysis, and the relevant checklist can be found as **S1 File** [20]. Between March 2012 and September 2013, periodic searches were performed in the following databases for potentially relevant studies: MEDLINE, Cochrane Central Register of Controlled Trials (CENTRAL), Cochrane Database of Systematic Reviews, Cochrane Methodology Register (CMR), Educational Resources Information Center (ERIC), Center for Reviews and Dissemination (CRD), ISI Web of Science and Google Scholar. Further, we searched the bibliographies of the included studies for other potential publications on the subject. Our search strategy included the following keywords in various combinations: medical student; medical students; undergraduate; medical; research; intercalated; bachelor; BSc; iBSc; theses; thesis; developing. The search strategy used for PubMed was as follows: ((((((medical student research) OR undergraduate research) OR medical thesis) OR intercalated bachelor) OR intercalated BSc) OR iBSc) OR undergraduate research developing.

**Inclusion criteria:** All study designs, including cross-sectional, prospective, retrospective and interventional studies, randomized controlled trials and qualitative studies.

**Exclusion criteria:** Studies containing inadequate information about the participants and type of study; studies in languages other than English; studies assessing outcomes unrelated to medical student research; theses or commentaries; studies aimed at postgraduates or undergraduates other than medical students; studies whose main population was MD/PhD students. Graduate-entry medical students, nonetheless, were *not* excluded from this review.

Two of the authors independently reviewed the studies that met these criteria and any disagreements were resolved by consensus. Basic data extraction tables were then used to extract the main finding and characteristics of each of the included studies. Quantitative studies (reporting odds ratios (OR's), p-values, percentages or other statistical measures) were separated from qualitative studies in order to improve the judgment of cumulative evidence.

Qualitative studies were included in order to help contextualize the quantitative outcomes and to provide insights and entry points for future research. Qualitative studies were defined as those studies which satisfied the following criteria: a) Their aims did not include the extraction of quantitative outcomes and thus did not perform any statistical analysis; b) They present original research with clearly-defined study populations; c) They utilize qualitative research methods, including semi-structured and unstructured interviews, open-ended survey questions, focus groups and examination of records and documents.

An integrated methodology was utilized to assimilate quantitative and qualitative outcomes into a single mixed-methods synthesis [21,22]. After relevant studies have been identified, a thematic analysis was performed. The literature search and article inclusion/exclusion strategy was aimed at retrieving all articles relevant to the subject of medical students' research, without prior conceptions or theories about expected outcomes. Hence, our thematic analysis was data-driven (as opposed to being theory-driven) [22]. Quantitative and qualitative outcomes were discussed together under relevant thematic subject headings.

Two types of quantitative outcomes were used for meta-analysis: percentages (for explorative outcomes) and odds ratios (for interventional/associative outcomes). Whenever relevant or needed, the corresponding authors (or, if unavailable, other authors) of included studies were contacted to get the raw data needed for meta-analysis. In some cases, other outcomes beside the ones mentioned in the original paper were identified in the raw data and used for the meta-analysis.

Further details about the methodology used in this paper, including outcome-specific quality assessment, statistical methods used and the strategy used to tackle study heterogeneity and potential publication bias can be found in our supporting information (**S2 File**).

## Results and Discussion

**Fig 1:**
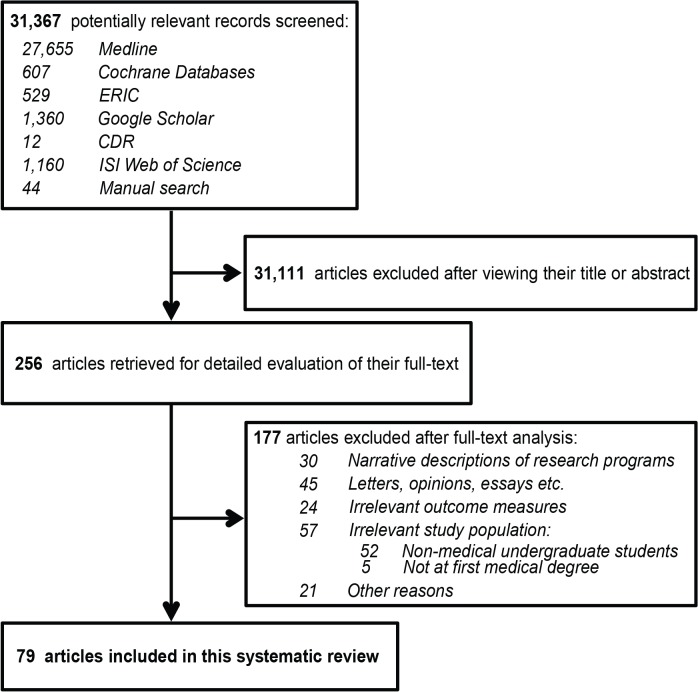
Flow diagram of the citation screening and article selection process followed in this systematic review.

Our search returned 31,367 records in the various databases. After reviewing the abstracts, 31,111 were excluded because they were either duplicates in various databases or satisfied one or more of the exclusion criteria mentioned earlier. 256 articles met (or were suspected to meet) our inclusion criteria upon reviewing their abstract and were thus retrieved for full-text assessment. Eventually 79 articles were found to match the selection criteria and were included in this review. More details about the article selection process can be seen in **Fig 1.**

Of the 79 articles retrieved, 71 were of quantitative nature, seven were of qualitative nature and one had both quantitative and qualitative components. Fifty-two articles were self-reported questionnaire studies with response rates ranging from 7.9% to 100%. Ten survey-based articles had response rates less than 60%. Twenty-three studies used a more objective research strategy that relied on searching institutional databases and records, two used both questionnaires and objective database searching and two had an unknown/undisclosed methodology. There were 47 cross-sectional studies, 25 retrospective studies, three prospective studies, three intervention studies and one study with an unknown/undisclosed design. Fifty-seven studies were performed in a single institution (including four qualitative study) and 22 studies involved multiple institutions (including four qualitative studies). Further, there were 14 studies that reported the effects of certain research programs or initiatives, whose study population might or might not be affiliated with multiple institutions. Sixteen studies assessed the value of intercalated BSc's (iBSc's) and 14 studies were carried out in developing countries.

**Fig 2:**
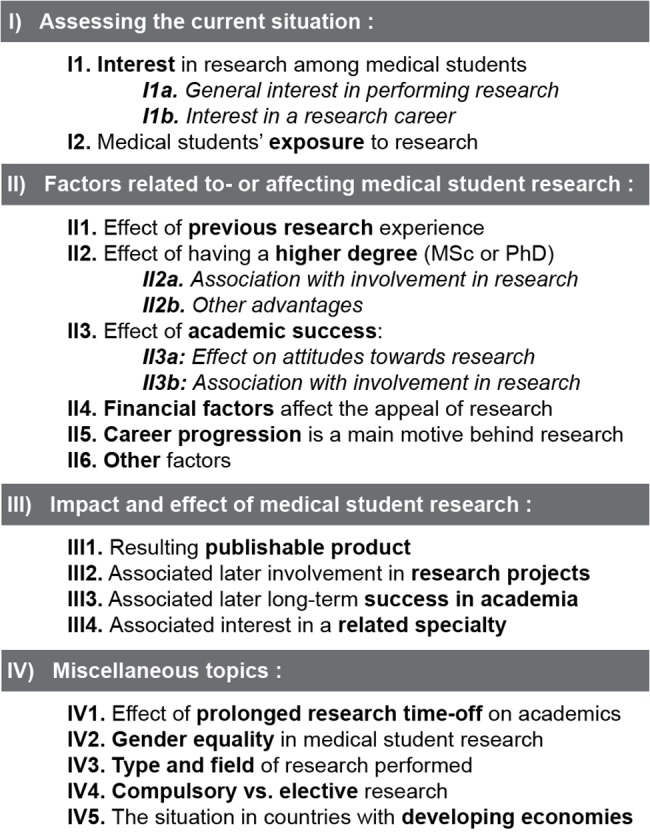
Themes and sub-themes resulting from the thematic analysis of included quantitative and qualitative studies.

After thematic analysis was performed, the resultant themes and sub-themes, outlined in **Fig 2**, also served as the scaffold for writing this paper. The data extraction and quality assessment worksheet and the relevant sensitivity plots can also be found in the supporting information files (**S3**and **S4 Files**, respectively) [7,8,10,11,23–90].

### Assessing the current situation

**Fig 3:**
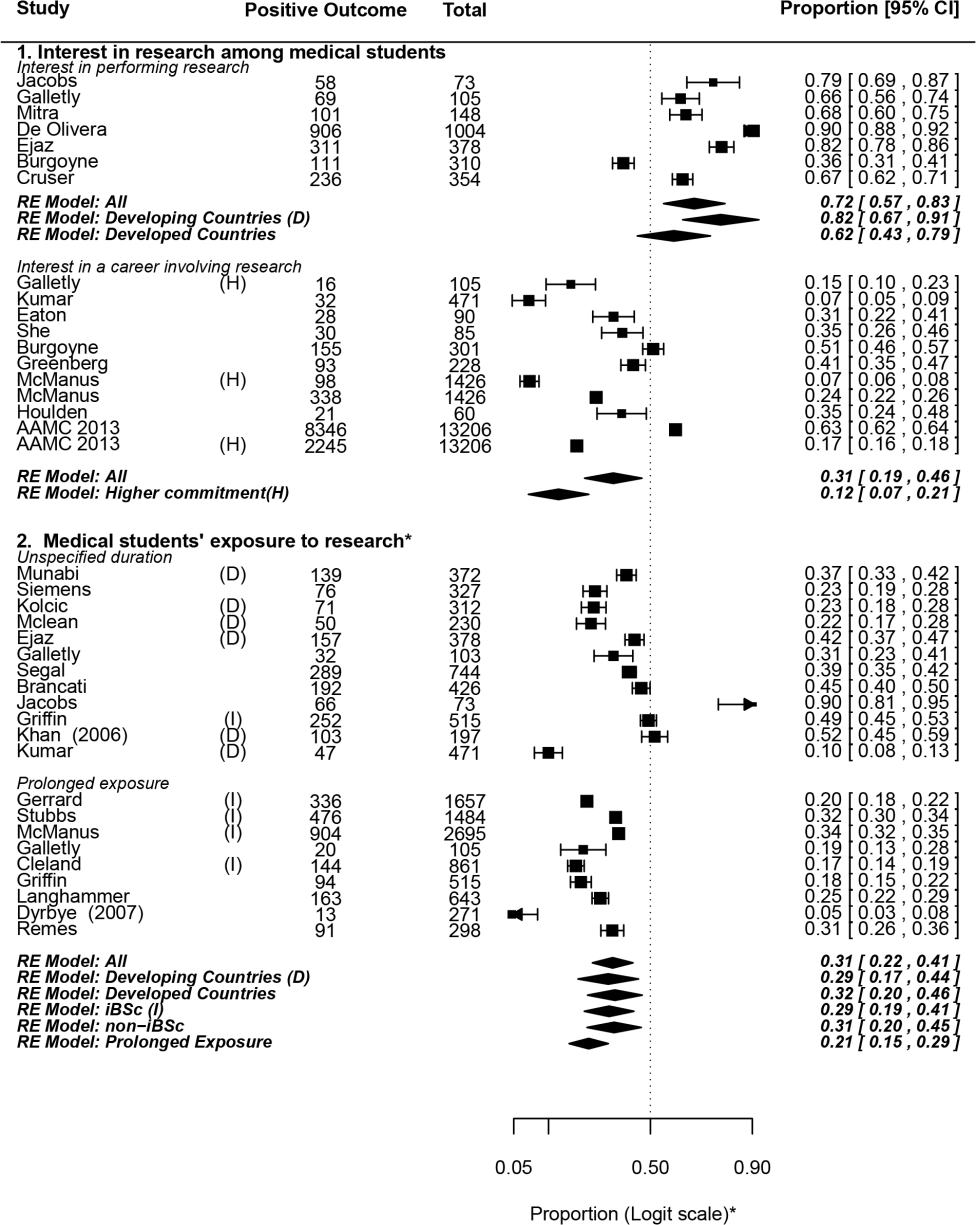
Assessing the current situation: Interest in- and exposure- to research among medical students. Forest Plot symbols: * The axis, not the data, is shown in logit scale for aesthetic purposes. Table symbols: * Mandatory exposure (in the form of curricular components or graduation theses) was excluded from this analysis. **Abbreviations used: D,** developing countries; **H,** higher commitment to a research career; **I,** intercalated Bachelor of Science degree (iBSc). Dates are shown beside studies that may be confused with others referenced in this review having the same similar first-author names.

We assessed the current state of medical student research by focusing on two main outcome measures: interest in- and exposure- to research among the medical student population. Both of these outcomes are explorative in nature (rely on proportions rather than odds ratios) and have been quantitatively pooled to yield a weighed estimate value. The results have been summarized in **Fig 3**[7,10,26,28,32,47–49,52,54,55,58,63,67–69,71–75,80–82,85,90–92]**.**

#### Interest in research among medical students

While the only reliable method for probing interest in medical research is assessing actual voluntary research involvement, survey data (self-reported interest) may provide insights into any discrepancies between interest and actual involvement. To avoid pooling survey data that are too heterogeneous, we made a distinction between survey questions that ask about general interest in research and those specifically asking medical students about their interest in making commitments to research during their future careers.

**I1a:** Interest in performing research: A pooled weighed estimate of 72% of medical students reported having interest in performing research (0.72, 0.57–0.83). One particularly high estimate was that reported by De Olivera and colleagues, which showed that 90% of its 1004 student sample had interest in performing research [74]. However, even when this study was excluded from the analysis as a possible exception, the pooled weighed estimate remained fairly high (0.67, 0.53–0.79) (**S4 File**).

**I1b:** Interest in a career involving research: The single best estimator of career intentions of US medical graduates is probably the Graduation Questionnaire (GQ), developed by The Association of American Medical Colleges (AAMC) in 1978 [7]. In 2013, 63% of the 13,180 respondents indicated intentions to become somewhat-to-exclusively involved in research during their medical careers, including 17% who planned "significant" or "exclusive" future involvement. This huge sample size approaches a true census, with 81.8% of the US fresh medical graduate population being covered.

Upon quantitative pooling of our included studies, we found that about 31% of medical students (0.31, 0.19–0.46) were interested in a career involving research, and 12% (0.12, 0.07–0.21) showed interest in "significant" (higher) commitment to research during their future careers. One particularly important, high-quality study was that of McManus and colleagues, showing that 6.9% of UK medical students planned to pursue academic careers (or found them to be very appealing) [85]. When we calculated the pooled outcome excluding MacManus et al or the AAMC data, the pooled proportion was not markedly changed (**S4 File**).

It should be noted that there is considerable variation in the proportions reported in our included studies. This may reflect inherent (true) variability in students' research interests due to diversity of settings and study populations (as has been discussed in **S2 File**). We also believe that there are other potential contributors to this variability, most notably the ambiguity of wording of survey questions. For example, many studies did not make a clear distinction between interest in an academic (university faculty) medicine career, and interest in a career involving some research outside of academia.

#### I2. Medical students’ exposure to research

Even today there is no consistent way in which undergraduate medical students are incorporated into research. For example, students may be engaged in research through summer research electives [9,45], mandatory curricular study modules [90], extracurricular research activities [93], or they might decide to intercalate for one or more years to obtain a BSc beside their medical degree. In Germany, it is mandatory for medical students to submit a thesis outlining the results of a research project in order to graduate with the title "Doctor" [30]. This requirement has also been reported in Peru, Finland, France and some U.S. universities such as Yale [24,27,76,94]. The AAMC 2013 Graduation Questionnaire shows that 68.2% of US medical graduates participated in a research project with a faculty member on a mandatory or volunteer basis and 41.7% co-authored a research paper [7].

If we exclude papers describing medical schools asking for mandatory graduation theses or research modules, we find that a little less than one third of medical students participated in research projects (0.31, 0.22–0.41). The proportion exposed to “prolonged” periods of research (>6 weeks) is even less (0.22, 0.16–0.28).

In the U.S., different medical schools have different research expectations, and the exposure of medical students to non-mandatory research seems to be largely dependent on medical school influence. Duke University, for example, incorporates students into summer-long research projects [95]. On the other hand, Stanford University, the University of Pittsburg and Warren Alpert Medical Schools incorporate students into longitudinal research projects in parallel with their academic studies [95–97]. This longitudinal approach may help in solving some of the reported problems of time-out research, such as the reluctance of medical students towards detachment from their colleagues and financial worries about spending extra time in college. Indeed, the success of Stanford is particularly evident, with 90% of medical students participating in research projects [91].

We found that the pooled proportion of medical students reporting some interest in research is higher than that of students who were actually involved in research projects. This may be due to: a) self-reported interest may not necessarily reflect serious willingness to pursue research; or b) lack of opportunities to meet students’ interest due to lack of funding, supervision and encouragement or inflexible curricula that leave little or no time for research (**S5 File**) [45,47–50,52,55,57,68,74].

### II. Factors related to- or affecting medical student research

**Fig 4:**
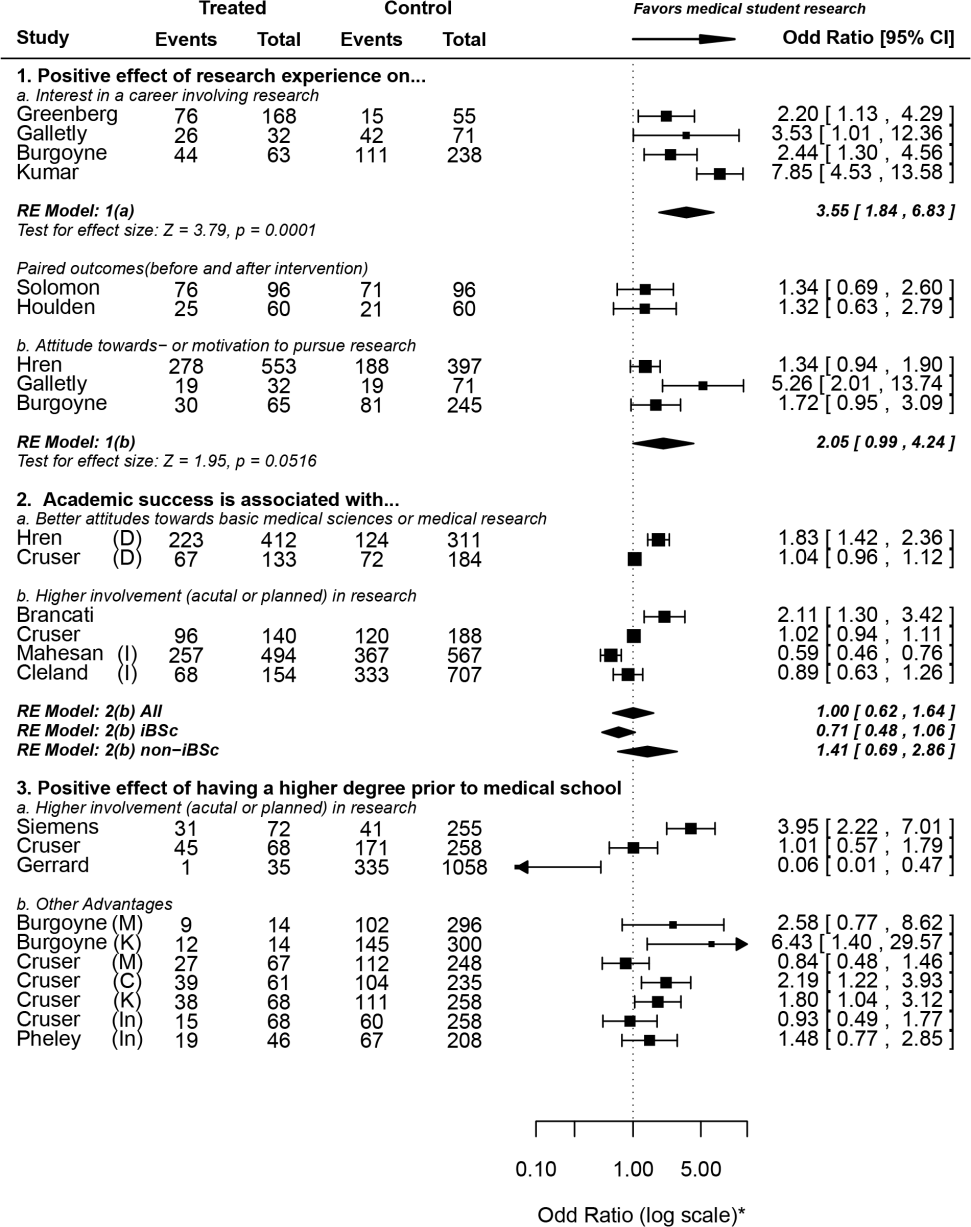
Factors related to- or affecting medical student research (i)–Effects of previous research experience, academic success and higher degree graduate-entry into medical school. Forest Plot symbols: * The axis, not the data, is shown in log scale for aesthetic purposes. **Abbreviations used: D**, developing countries; **I**, intercalated Bachelor of Science degree (iBSc); **M**, motivation to perform research; **K**, research knowledge or skills; **C**, confidence in research competencies; **In**, interest in research. For some studies, odds ratios and 95% confidence interval values were reported, but not the raw numbers.

**Fig 5:**
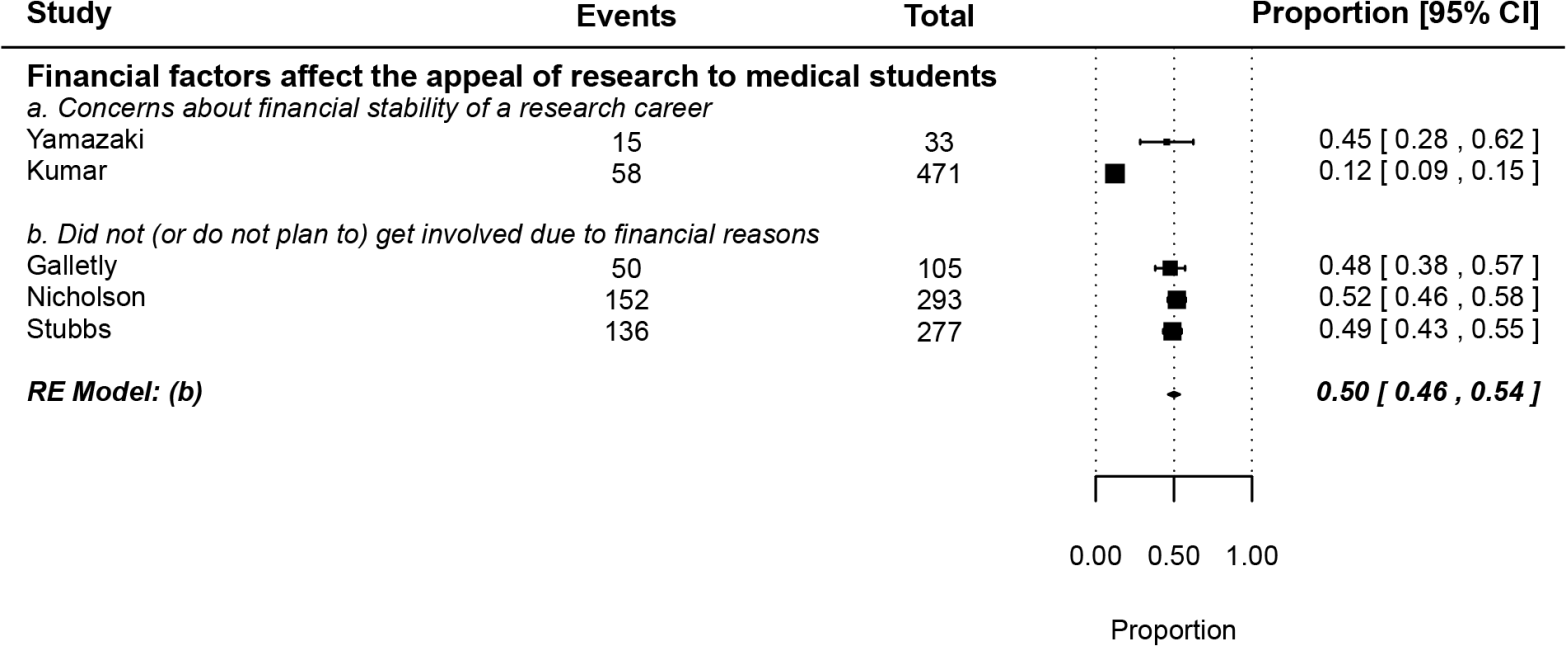
Factors related to- or affecting medical student research (ii)–The effect of financial factors on appeal of research to medical students.

We identified four main factors affecting medical student research: previous research experience, academic success, having a higher degree (MSc or PhD) at the time of matriculation and financial factors. The effects of the first three factors were reported using odds ratios due to the presence of untreated groups (**Fig 4**) [32,47,52–55,58,62,63,67,79,81,92,98], while the fourth factor (financial influence) was pooled using proportions from survey studies (**Fig 5**) [55,57,59,67,82]. Moreover, we discuss the results of various studies reporting other relevant factors that could not be meta-analyzed, including the role of mentorship and competitive residencies in shaping medical students’ perceptions about- and attitudes towards research.

#### II1. Effect of previous research experience

Students who participated in research projects during medical school were over three times as likely to report interest in research involvement during their future careers (OR = 3.55, 1.84–6.83). Two studies [92,98], which were not included in the pooled weighed estimate, reported paired outcomes, with non-significant differences in research career interests after research exposure. Additionally, we found that medical school research involvement has no significant correlation with attitudes or motivation towards research (OR = 2.05, 0.99–4.24).

It is difficult to conclude that self-reported interest is a direct effect of exposure to research, since reverse causality cannot be excluded. That is, it is logical to assume that a fairly large proportion of students who had pre-existing interest in a career in research decide to participate in research projects. As a matter of fact, students in two of the included studies agreed that research participation strengthened *pre-existing* interest in a research career [90,91]. These findings also make sense in light of the fact that over half of all medical students reported having some interest in a career involving research (**Fig 3**). Another possible explanation for the above results is that students who have had prior research experience have better research knowledge and skills, and are therefore more confident about their ability to succeed were they to undertake research projects during their future careers. Indeed, in a series of interviews conducted by Jones et al, students who undertook an intercalated BSc in primary healthcare reported a positive influence of the experience on their appreciation of the research process [99]. Similarly, a thematic analysis of 905 SSC (Student Selected Component) projects by Murdoch-Eaton et al provided by medical students at six UK medical schools revealed gain of various research-related skills [90]. These results are also supported by eleven quantitative studies, summarized in **S5 File** [11,37,39,40,46,47,55,64,82,89,91].

#### II2. Effect of having a higher degree (MSc or PhD) prior to medical school

**II2a:** Having a higher degree is associated with involvement in- (or planned involvement in-) research: Siemens et al report that medical students who had a higher degree prior to enrolment in medical school were almost four times more likely to perform research during medical school (OR = 3.95, 2.22–7.01) [52]. However, data provided by Cruser et al showed no significant difference between the two groups regarding their planned involvement in future research (OR = 1.01, 0.57–1.79) and Gerrard et al actually reported the reverse trend, with higher degree graduate-entry medical students actually being *less* likely to pursue an iBSc [54,81]. This is consistent with data we obtained from Mahesan et al, which shows that graduate-entry medical students (having any degree prior to matriculation) were almost ten times *less* likely to pursue an intercalated degree (OR = 0.01, 0.00–0.13) [62].

Since career progress (especially the pursuit of competitive residency) is a major motive behind medical student research, it may be argued that medical students with a higher degree view this aspect of their Curriculum Vitae (CV) as being “complete enough” and hence devalue the pursuit of another degree. In fact, to the medical student with a prior degree, an iBSc will almost always result in degree duplication, even if the skills and knowledge base of the iBSc course were completely different from those of the other degree already gained by the student.

**II2b:** Other advantages of having a higher degree (MSc. or PhD.): There is no significant correlation between having a higher degree prior to medical school enrolment and research interest or motivation. However, as might be expected, higher degree graduate-entry medical students were more knowledgeable about research, showed better research skills and had higher confidence in their research competencies (**Fig 4**). This is expected, given that almost all higher degrees have a compulsory research component.

#### II3. Effect of academic success

**II3a:** Academic success is associated with attitudes towards basic medical sciences or medical research: The data we obtained from Hren et al shows an association between higher Grade Point Average (GPA) and attitudes towards research (OR = 1.83, 1.42–2.36) [79]. Cruser et al’s data, on the other hand, shows no significant difference between highest MCAT (Medical College Admission Test) scores and attitude scores [54]. Perhaps GPA *during* medical school, but not before admission, is a factor that influences attitudes. However, we believe the evidence in favor or against this hypothesis is weak and further investigation is needed in the future.

**II3b:** Academic success is associated with involvement in- (or planned involvement in-) research: The weighed pooled odds ratio from four included studies shows no association between academic success and involvement (or planned involvement) in research projects (OR = 1.00, 0.62–1.64). The only study showing a significant correlation was Brancati et al, which asserts that students who were academically successful (top third of their class) were more likely to choose an academic career (OR = 2.11, 1.30–3.42) compared to their less successful peers (lower third) [32]. However, this study investigates choice of an academic career rather than involvement (or planned involvement) in research during or right after medical school. Hence, it may be argued that this study should be excluded from the analysis as it measures a different outcome, in which case the pooled odds ratio remains non-significant (0.82, 0.59–1.15). We suggest further investigation into this issue using studies with more favorable, preferably prospective, designs.

#### II4. Financial factors affect the appeal of research to medical students

About half of medical students who chose not to get involved in research reported being deterred by financial factors (0.50, 0.46–0.54) (**Fig 5**) [55,57,59,67,82]. Nicholson et al and Stubbs et al both show that about half of medical students who choose not to intercalate do so for financial reasons [59,82]. In addition, Galletly et al also reported that about half (48%) of medical students asserted that perceived lower salaries of academicians was an important factor behind their decision not to pursue an academic career [55]. The consistency of the findings by the former two studies with the latter one suggests that it's not just the short-term financial burden of pursuing an intercalated degree that deters medical students from getting involved in research, but a general long-term financial concern. Financial worries, particularly the fear of running out of grant money and the financial stress of academic careers, were indeed cited by students interviewed by O'Sullivan et al among the deterrents to academic career pursuit [100].

Similarly, Yamazaki et al and Kumar et al both showed that a considerable fraction of the general medical student population displayed concerns about the financial stability of a research career (45% and 12%, respectively) [57,67].

#### II5. Career progression is a main motive behind performing research during medical school

The result from seven included studies indicate that career progression is a main motive (if not *the* main motive) behind performing research during medical school. These results indicate that in a large fraction of cases, medical students perform research for purely pragmatic reasons (related to their residencies or further post-graduate education), rather than pursuing research for the value it has in and of itself **(Table 1)** [48,49,52,54,55,82,86].

**Table 1:** Career progression (eg securing competitive residency) is a main motive for medical students to perform research.

Study	Type	Design	Control group	Institutions	Lim.	Overall quality	N	Population	Outcome measure	Outcome
**Siemens**	R/X	Q	Y	M	Rp	Medium	327	Second- and Fourth- year medical students; Three Canadian Medical Schools.	Seeking competitive residency correlated with research activity	P<0.001
			N	M					Got involved in research to facilitate admission into residency	140 (42.8%)
**Cruser**	R/X	Q	Y	S		Medium	354	Incoming, first, and second year classes of osteopathic medical students at University of North Texas Health Science Center; Research competencies.	Agree that "to be accepted into competitive residency, I have to have some knowledge or experience in research"	226 (63.4%)
Griffin *†	X	Q	N	M		Medium	72	Students; seven medical schools in UK.	Were mainly motivated to publish for career progression.	37 (51.4%)
Remes **	X	Q	N	S	Rp.	Medium	91	Students; University of Helsinki	Securing residency main reason behind performing research	79 (87%)
**Baig ‡**	X	Q	N	M		Medium	398	Medical students who showed interest in medical research; Four Medical Schools, Karachi, Pakistan	Seeking residency in the US main motive to perform research	159 (39.9%)
									"Desire for a strong CV" main motive to perform research	275 (69.1%)
Stubbs **†	R	Q		M	Rp.	Medium	253	Students who chose to intercalate; Bristol and Sheffield Medical Schools, UK	Did an intercalated degree "to get the job they want"	176 (69.6%)
**Galletly**	X	Q	Y	S		Medium	98	Final year students; University of Adelaide.	Students seeking higher degree (MSc or PhD) more likely to perform research during medical school **§**	OR = 3.17 (1.09–9.18)
							100		Students seeking higher degree (MSc or PhD) more interested in research **§**	Or = 5.68 (1.2–27.0)

* Baseline population is students who submitted an article for publication

** Baseline population is students ho performed research

**†** Studies assessing the effect of an Intercalated Bachelor of Science (iBSc)

**‡** Study performed in a developing country (Pakistan)

**§** Adjusted for age, sex and interest in a career involving research (data obtained directly from authors and dichotomized).

**Abbreviations used: X,** Cross-sectional; **R,** Retrospective; **Q,** Questionnaire; **Y,** Yes; **N**, No; **S,** Single; **M,** Multiple; **Lim,** Other limitations; **Rp,** low response rate (<60%).

Four studies mentioned the role competitive residencies play in driving medical students to perform research, and in fact students in three of those studies believed that seeking competitive residency was–explicitly- the main motive to perform research during medical school. The results from a qualitative study by Shapiro et al support this conclusion by showing that the motives behind research participation include (but are not limited to) pragmatic targets such as improving the students' relationship with faculty [101].

These conclusions are consistent with other results reported here showing that: a) there is a discrepancy between interest in clinical practice and interest in a research career **(S5 File)** [45,51,56,57] and b) there is a correlation between interest in academia or basic medical sciences and interest in research **(S5 File)** [55–57].

Combined, these findings indicate that any policies aimed at boosting medical students’ engagement in research have to align research involvement with the career progress and success of students. In much the same way that peer-reviewed publications are a key competitive edge in academia and in competitive residency applications, it must become clear that research is more than just an accessory when it comes to ordinary clinical practice.

#### II6. Other factors related- to or affecting medical student research

As Reynolds has discussed, it is simply not enough to match students with professors in research projects, as good quality research requires *real* mentorship [102]. Research instructors also act as role models to encourage students to pursue careers in academic medicine. Further, finding the right mentor is important to ensure that students provide a working *and intellectual* input into the research projects, rather than simple assistantship in lab work or data collection **(Table 2)** [48,52,57,58,82].

**Table 2:** Positive effect of mentorship or the presence of an academic role model.

Study	Type	Design	Control group	Inst.	Lim.	Overall quality	N	Population	Outcome measure	Outcome
**Siemens**	X/R	Q	Y	M	Rp	Medium	327	Second- and Fourth- year medical students; Three Canadian Medical Schools.	Mentorship bolsters interest in research	P = 0.05
									Role models drive interest in academia	P = 0.015
Stubbs *	R	Q	Y	M	Rp	Medium	1484	Students who chose to intercalate; Bristol and Sheffield Medical Schools, UK	Students with clinical academic supervisors gained significantly more publications	P<0.0001
									Students with clinical academic supervisors gained significantly more posters	P = 0.0002
									Students with clinical academic supervisors gained significantly more first-class honors	P = 0.055
**Greenberg**	R	Q	Y	M	Rp	Medium	228	Third and Fourth year students; Three US Medical Schools	Mentorship bolsters interest in a research-oriented career **	OR = 2.5 (1.39–4.51); P = 0.002
**Yamazaki**	X	Q	Y	S	Rp	Medium	267	Students; Juntendo University School of Medicine, Japan	Interest in basic sciences correlated with faculty's efforts to promote interest in research.	OR = 2.86(1.62–5.06); P = 0.0003
**Griffin** *	X	Q	N	M		Low	72	Students; seven medical schools in UK.	Were encouraged to submit paper by supervisor	7 (9.7%)

* Studies describing the effect of an intercalated Bachelor of Science (iBSc)

** Data obtained directly from authors and dichotomized.

**Abbreviations used: X,** Cross-sectional; **R,** Retrospective; **Q,** Questionnaire; **Y,** Yes; **N**, No; **Inst.**, Number of institutions; **S,** Single; **M,** Multiple; **Lim,** Other limitations; **Rp,** low response rate (<60%).

This is not always going to be easy; the results from two qualitative studies show that the complexity of ethical approval procedures (whether in terms of time or paperwork) is a major difficulty facing supervisors and students alike [90,103]. Further, the absence of clear, well-structured research governance may result in some aversion to faculty-mentored student research. This was the case in two qualitative studies, where students cited problems with approachability of faculty members and expressed concerns about being used as "free labor" on research projects [90,101].

In fact, Murdoch-Eaton et al's aforementioned project content analysis, while revealing some gain in useful research skills, also highlighted the failed attainment of a *balanced* skill-set; the majority of student projects involved information gathering and data processing, while fewer projects involved actual student engagement in research methodology development or critical analysis of data [90].

It may be presumed that the relatively short duration of the undergraduate research experience could limit its publication or citation potential. Indeed, Dyrbye et al found that graduates with a 17–18 week-long research experience published significantly less papers in which they appeared as first authors than their peers who spent 21-weeks doing research [29]. Further, Fede et al showed that the annual Undergraduate Medical Congress of ABC foundation (COMUABC) had a smaller proportion of abstracts accepted for publication in peer-reviewed journals in comparison to conferences of practicing physicians [70]. Conversely, Van Eyk et al. reported that the average number of citations of Dutch medical student publications was actually *higher* than the average citations for papers in the same field. [41]

A number of studies investigated factors that prevent medical students from being involved in research. Poor mentorship, lack of role models and perceived lower salaries of academic physicians were among the key factors cited **(S5 File)**. The previous findings were also supported by four qualitative studies **(Table 3)** [17,45,90,99–101,103,104]**.**

**Table 3:** Studies with a qualita3tive component.

Study	Type	Design	Study population and setting	Respondents (Response rate)	Outcome measures	Outcomes
**O'sullivan**	X	IN	Students, residents and faculty members; University of California, San Francisco.	40 (11 medical students)	Factors related to pursuit of academic medicine career.	Early exposure to research, finding the right mentors and role models are among the most important factors. Sociocognitive factors such as financial worries play a role.
**McGee**	X	IN; GTM	Participants; SURF program at Mayo Clinic College of Medicine + Participants; IMSD program at Mayo Clinic; 1997–2000.	109 (20%)	Themes related to pursuing a PhD, MD/PhD or MD with a research intention.	Five major themes are relevant: Curiosity, Problem solving, independence, serving the world indirectly and a flexible perspective of one's own future. MD-bound students talked about a desire to help others *directly* through patient care, while MD/PhD or PhD-pursuing students wanted to help others *indirectly* through research.
**Shapiro**	Ret	GTM	Participant students and mentors; Summer research assistantship program in family medicine.	11 students 10 faculty mentors	Motives behind participation	Most students were driven by curiosity, the will to learn about research and to improve their relations with faculty. Faculty mentors wanted to be more involved with the students and to attract more of them into research.
**Murdoch-Eaton**	X	Mixed: SG; FG; PA.	Students; Hull, York, Leeds, Liverpool, Newcastle and Sheffield Medical Schools.	**Focus groups:** 5 groups (one per school) **Study groups:** 15 students (three per school). **Projects:** 905	Perceptions about undergraduate research and thematic analysis of Student Selected Component (SSC) projects.	**Focus groups:** students understood the benefits of research learning and skills, but mentioned practical difficulties. **Study groups:** confusion between research and clinical practice among students. **Project analysis:** Various skills research-related skills were gained by students.
**Sanchez**	X	FG	Students; Three national student conferences in the US: Interest in an academic medicine career.	73	Interest in academic medicine careers.	Lack of knowledge or competency cited as obstacles in pursuing academic medicine career. Mentorship and career development resources cited as potential improvement strategies. Higher involvement of ethnic minorities prompted.
**Pacifi**	X	Mixed: Q; IN; CS	Undergraduate Upper-level science major premed and non-premed students; Southeastern United States.	135 (7.9%): *55 Premed 80 non-premed*. 11 interviews	Influences and experiences regarding undergraduate scientific research	**Premeds:** talked more about serving humanity and the empathic aspects of research, viewed it as a tool to augment their professional outlook but not as a career. **Non-premeds**: talked more about the joy of discovery and were enthusiastic about a career in research. **Both groups**: had similar expectations from research.
**Jones**	X	IN	Students who did an intercalated BSc in primary Healthcare.	24 (92%)	Perceptions and outcomes.	Greater awareness about research and critical appraisal. More informed career decisions. Deeper insights into the psycho-cognitive aspects of illness.
**Robinson**	X	IN	Academic supervisors and administrative staff; three UK medical schools.	12	Impact of research governance on research education.	Ethical approval bureaucracy cited as a main limiting factor. Supervisors tend to avoid them by modifying existing projects or abandoning supervision altogether.

Abbreviations: **X**, cross-sectional; **Ret**, retrospective; **Q**, questionnaire; **IN**, interview; **GTM**, grounded theory methodology; **SG**, student groups; **FG**, focus groups; **PA**, project analysis; **CS**, case study.

In addition, institutional influence as well as the type and length of available research opportunities were found to be relevant factors in determining whether students choose to engage in research [51,53]. McLean and co-authors provided an excellent set of tips to bolster the involvement of students in academic medicine projects and potentially overcome some the aforementioned limitations [105].

. The importance of psycho-cognitive factors in determining medical students' motivation towards- and engagement in- research was also highlighted in the qualitative literature. One of the most important motives behind performing research is curiosity. Not only is curiosity a main motive behind pursuing research while in medical school (as has been shown by Shapiro et al [101]), it is one of the very early psycho-cognitive predictors of persistence into scientific or research disciplines even before enrolment into medical school [17,104]. Conversely, perceived lack of competence may deter medical students from pursuing research-active careers [45].

### III) Assessing the impact and effect of medical student research

**Fig 6:**
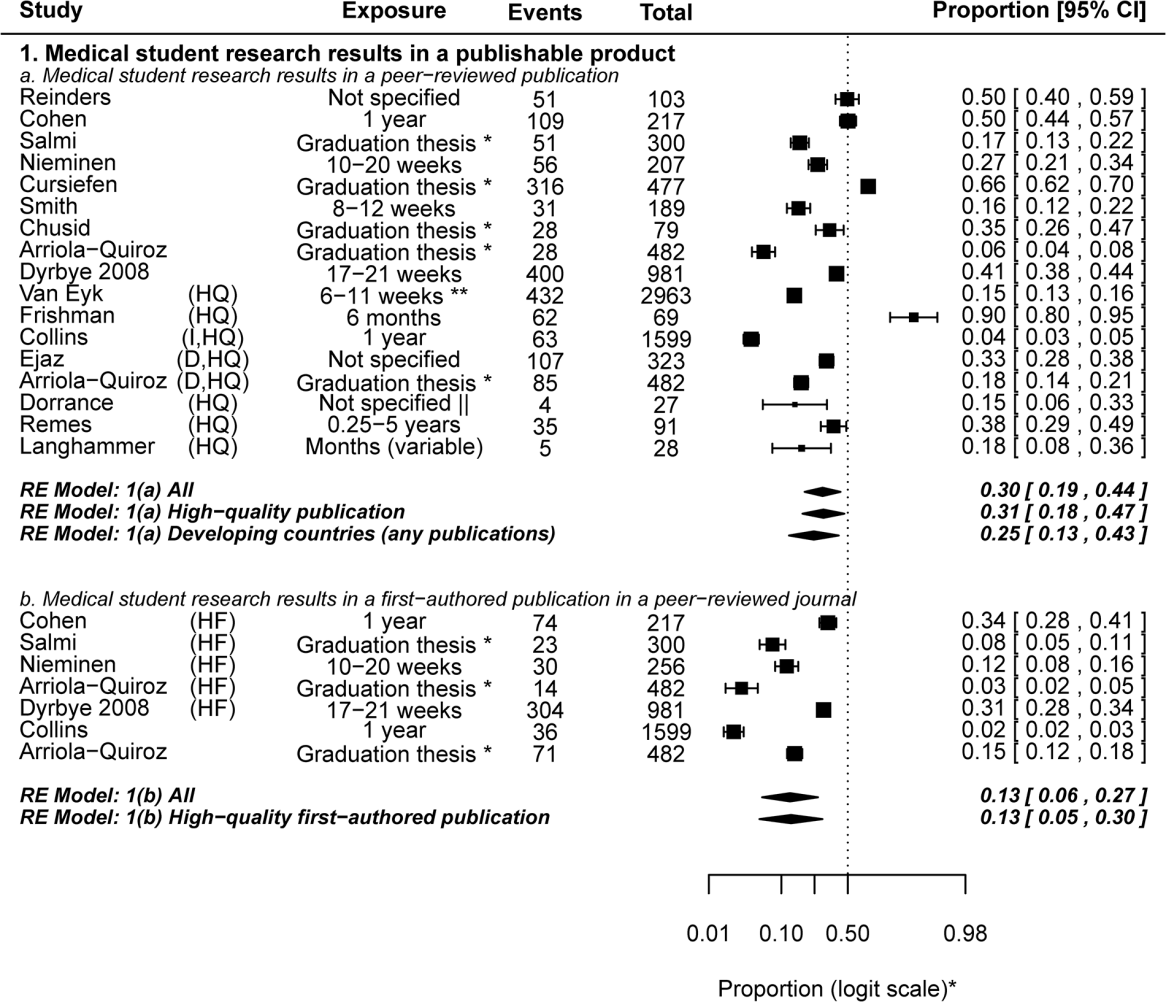
The proportion of medical student research resulting in a peer-reviewed journal publication. Since the duration of research exposure will almost always affect the publication outcome, it has been shown too. Forest Plot symbols: * The axis, not the data, is shown in logit scale for aesthetic purposes. Table symbols: * The duration is probably prolonged (possibly months long); ** 20–40 European medical school credits; **||** For published projects, the average duration was 18 months. **D**, developing countries; **I**, intercalated Bachelor of Science degree (iBSc); **HQ**, relatively high quality publication (indexed in Medline, Scopus or Medic), **HF**, first-author publication in a relatively high quality journal. Dates are shown beside studies that may be confused with others referenced in this review having the same similar first-author names.

**Fig 7:**
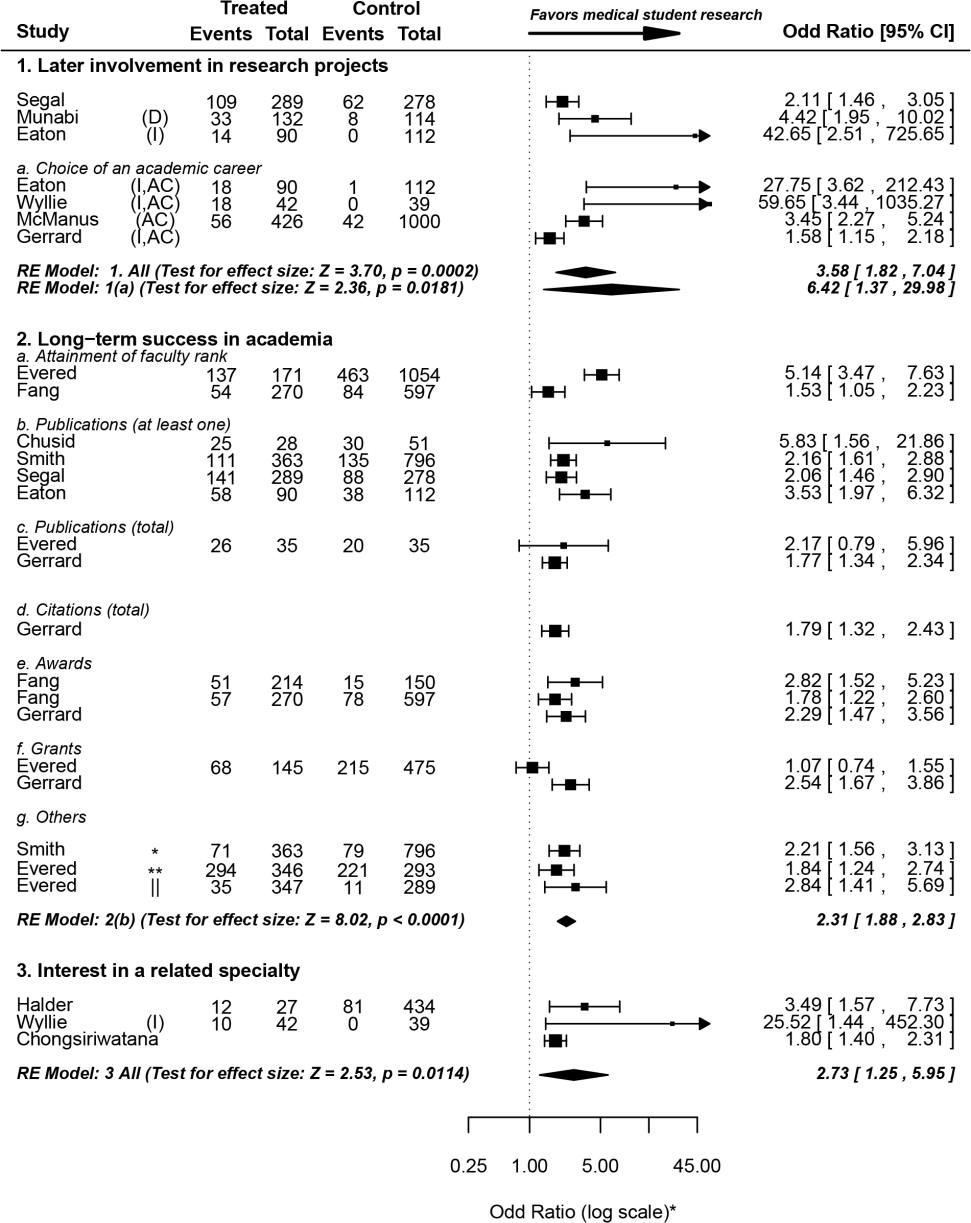
The impact of medical student research–Impact of medical student research on career choice and long-term academic success. Forest Plot symbols: * The axis, not the data, is shown in log scale for aesthetic purposes. Table symbols: * at least one first-author publication; ** at least one citation; **||** more than 20 citations. For some studies, odds ratios and 95% confidence interval values were reported, but not the raw numbers.

We assessed three main outcomes that reflect the short- and long- term impact of medical student research: 1) the proportion of research performed during medical school that culminates in a peer-reviewed journal publication, 2) the effect of medical school research on the career choice and future research involvement of medical students, and 3) the effect of medical student research on long- term success in academia. The first outcome has been summarized in **Fig 6**[10,24,25,27,29–31,37,38,41,49,64,75,76,93,106] and the latter two are shown in **Fig 7**[8,25,26,31,43,44,66,68,81,83,85,90].

#### III1. Medical student research results in a publishable product

Peer-reviewed journal publications are generally considered to be the best indicator of research productivity, and it may be viewed as a major metric (though not the only one) of the “return on investment” in supporting and funding medical student research. An average of 30% (0.30, 0.19–0.44) of research performed by medical students resulted in a peer-reviewed journal publication. When only higher quality publications were included in the analysis (indexed in Medline, Scopus or Medic), the proportion remained more or less the same (0.31, 0.18–0.47). Subgroup analysis of studies investigating the research productivity of graduation theses revealed that 26% (0.26, 0.10–0.52) of graduation theses result in higher quality publications.

As expected, all studies reporting *first*-authored peer-reviewed publication by medical students described instances of prolonged research exposure. An average of 13% (0.13, 0.06–0.27) of medical student research resulted in a first-authored peer-reviewed publication. The pooled outcome remained the same when only higher quality publications (Medline-, Scopus- or Medic- indexed) were included in the analysis (0.13, 0.05–0.30).

A few initiatives, aimed at propping up medical student publication output, have gained popularity over the last few years. Those initiatives include a number of student-run journals and journal spaces dedicated solely for medical student research publications [107–110]. A subset of these journals is Medline-indexed and some even involve undergraduates in the peer-review process. Similarly, the Yale Journal of Biology and Medicine annually publishes Yale's student thesis abstracts [111]. These initiatives, we suppose, will help in promoting student participation in research and comfort students about publication issues. To our knowledge, there is no systematic investigation in the literature so far regarding the quality of research published in medical student research journals in comparison to field-specific journals. Hence, we would like to take a conservative stance whenever we see such hierarchical "segmentation" of the scientific enterprise; the stringency of research assessment, in our opinion, should be indiscriminant to the identity of the study authors.

It is important to note that the failure of publication of medical student research may be reflective of other factors beside the success and relative contribution of the student. For example, Weber et al showed that 55% of the papers submitted to a medical specialty conference did *not* reach the stage of publication five years later [112]. Similarly, Riveros et al found that half of the clinical trials reporting results in ClinicalTrials.gov had no corresponding journal publication [113]. Keeping this in mind, the results by Cursiefen et al should not be surprising; showing that medical students were among the authors of 28% of the papers produced by a German medical faculty, even though only 66% of medical student research resulted in a publication [30].

#### III2. Research during medical school is associated with later involvement in research projects

Students who took part in research projects during medical school were more likely to get involved in (or report planned involvement in-) research later in their careers (OR = 3.58, 1.82–7.04). When a subgroup analysis was performed to include only studies that explicitly refer to academic careers (as opposed to brief research encounters), students who performed research during medical school were over six times as likely to pursue academic careers (OR = 6.42, 1.37–29.98) than their “untreated” peers.

With one exception, none of the included studies had a prospective design; hence reverse causality cannot be excluded, and is in fact very likely (students planning academic medicine careers choosing to get involved in research during medical school). Indeed, the only prospective study included (McManus et al [85]) showed that at the time of application to medical school, students who later chose to take an intercalated degree were already significantly more likely to report definite or highly likely choice of academic medicine careers (OR = 1.37, 1.13–1.66). Just before graduation, however, this likelihood had a substantial increase (OR = 3.45, 2.27–5.24). Together, these results indicate that medical school research strengthens pre-existing interest in an academic career.

A qualitative study by O'sullivan et al emphasized the value of early research exposure in giving medical students the opportunity to entertain the thought of pursuing academic careers [114]. Such exposure, they concluded, may sometimes even *discourage* students from pursuing academia, but is necessary nonetheless given the lack of sufficient free time during post-graduation residency to experience research.

#### III3. Research during medical school is associated with long-term success in academia

Three studies showed that physicians who performed research during medical school were more likely to attain faculty rank long after graduation [8,32,66]. While this has implications on the decision of individual medical students to pursue research, we argue that it has little bearing on policy decision-making, since faculty positions are awarded on a competitive basis. Indeed, Brancati et al showed that this effect was dependent on the publication status of research performed during medical school [32]. In other words, students who did not publish their research were not significantly more likely to attain higher faculty rank on the long run. Hence, the fact that medical student research is associated with higher likelihood of attaining faculty positions has little implications regarding the systematic incorporation of research into medical curricula.

Students who performed research during medical school were more than twice as likely to author at least one peer-reviewed publication later in their career (OR = 2.31, 1.88–2.83). This remained true after the exclusion of Chusid et al [25] (which correlates successful publication of graduation theses with long-term publication success) from the analysis (OR = 2.26, 1.83–2.77). They were also twice as likely to acquire first-authorship (OR = 2.21, 1.56–3.13). The *total* number of publications and ability to secure grants, too, was reported to be significantly higher among students with medical school research experience [81]. Evered et al, on the other hand, found no significant difference in either of those measures between both groups [66]. Moreover, students who performed research during medical school were more likely to be cited at least once [66], had a higher total citation count [81], were more likely to be cited more than 20 times [66], and had higher odds of receiving awards [8,81] later in their careers.

While this data provides strong evidence of a correlation between medical school research and long-term success in academia, a causal relationship cannot be established since students who decide to perform research may already have a keen interest in research. Nonetheless, a causal relationship is quite likely since early research experience (especially if it culminates in a first-authored publication) would naturally enhance the career prospects and significantly improve the CV’s of early career medical graduates. Overall, we believe that the long-term impact of medical school research is inadequately assessed, and that further evidence is needed using prospective study designs with proper adjustment for baseline status.

#### III4. Research during medical school is correlated with career choice of- (or interest in a career in-) the same or related specialty as the research project

Three of the studies that met the broad inclusion criteria reported results from control or “untreated” groups. Other studies reported results only from treated groups and hence were excluded from the analysis. Overall, students are 2.7 times as likely to be interested in careers in the same (or related) clinical specialty as the research project they got involved in during medical school. As with many other conclusions in this review, a causal relationship cannot be determined from this apparent correlation. This is especially true in the case of competitive residencies (and is particularly relevant to US residencies), where research experience in the same specialty gives recent graduates a competitive edge over their peers without such experience.

The relationship between medical school research and clinical practice was also touched upon in two of the included qualitative studies. Shapiro et al showed that many faculty members mentored student research in family practice *in order to* attract students to the same specialty [101]. Indeed, students interviewed by Jones et al believed an iBSc in primary healthcare provided them with deeper insights into patient care and a more thorough understanding of evidence-based clinical practice [99].

### IV) Miscellaneous topics related to medical student research

**Fig 8:**
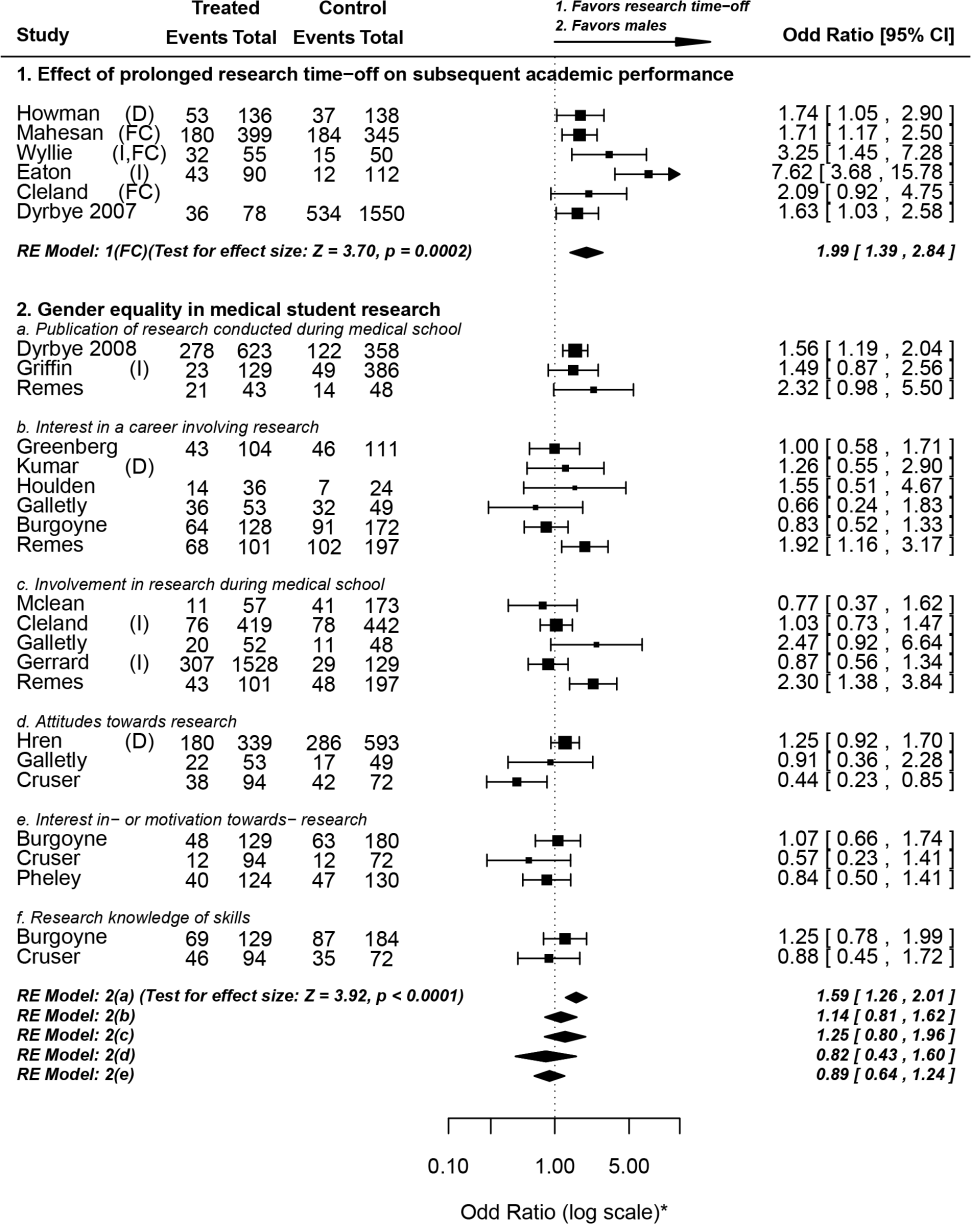
Miscellaneous topics related to medical student research. Forest Plot symbols: * The axis, not the data, is shown in log scale for aesthetic purposes. **Abbreviations used: D,** developing countries; **I,** intercalated Bachelor of Science degree (iBSc); **FC**, studies measuring final year academic performance and controlling for baseline performance. Dates are shown beside studies that may be confused with others referenced in this review having the same similar first-author names. For some studies, odds ratios and 95% confidence interval values were reported, but not the raw numbers.

**Fig 9:**
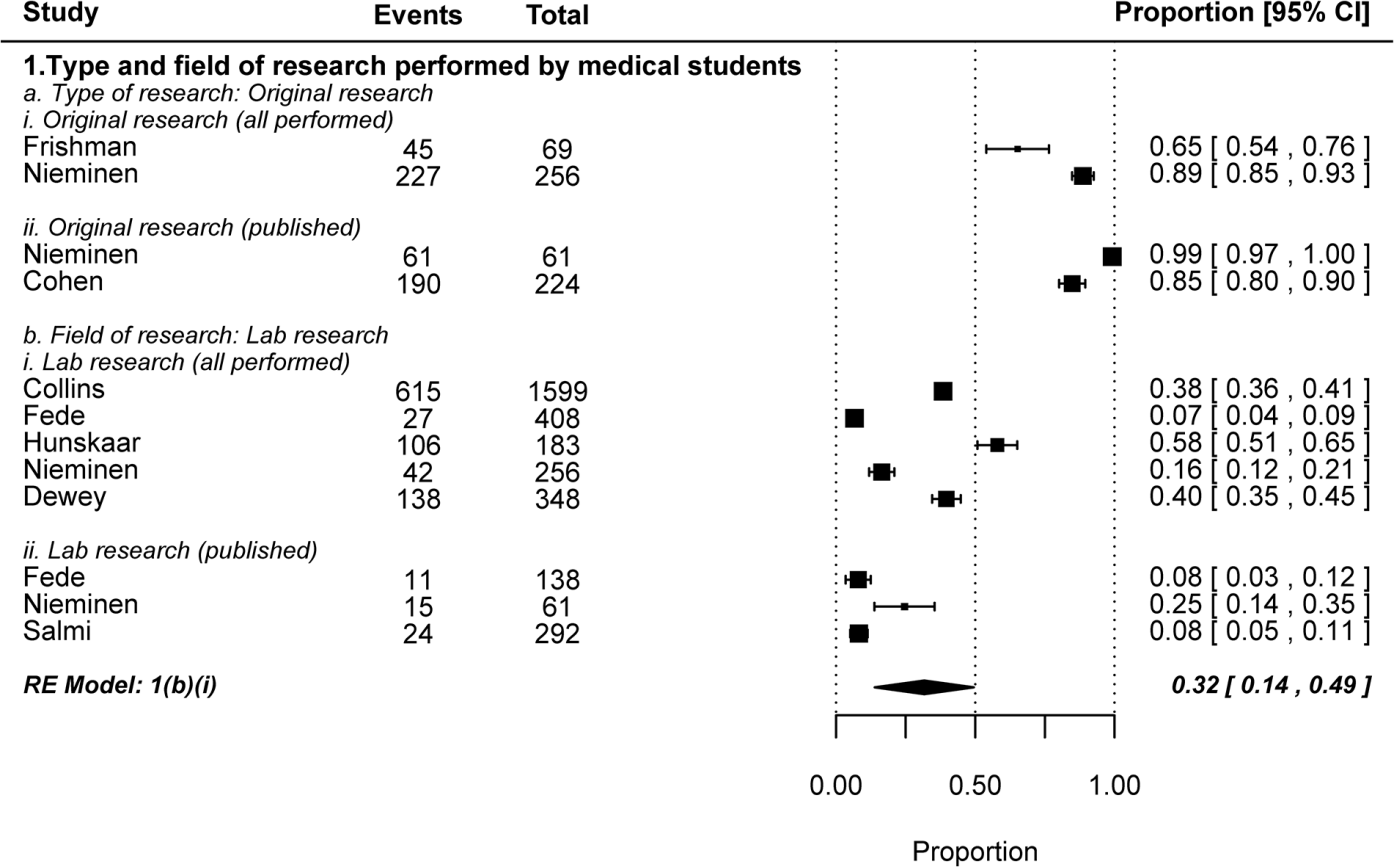
Characterizing the research performed by medical students.

In the following section of this review we discuss a number of miscellaneous topics relevant to medical student research. Three of these topics were discussed in light of quantitative data, and are summarized in **Fig 8**[28,29,47–49,53,54,58,59,62,63,67,71,79,81,83,88,89,92] and **Fig 9**[24,27,33,37,38,50,64,70]. Though they did not pass our inclusion criteria, four of the citations screened were personal perspectives provided by medical students, and are worth mentioning for enriching the discussion. They discussed the importance of the research experience on their medical career [115,116], the importance of medical students' research in increasing national research output [117] and the relevance of lab research involving animals to appreciation of human anatomy and physiology [118].

#### IV1. Effect of prolonged research time-off on subsequent academic performance

One of the issues discussed in the literature is the effect of prolonged research time-off amid the medical program on subsequent clinical knowledge. This question has been assessed in the context of iBSc degrees in a recent review [119]. All but one of our included studies investigated the effect of taking an intercalated degree on subsequent academic performance. The results have been conflicting; two studies that either matched groups by previous performance or adjusted for pre-clinical scores found no evidence of improvement in scores [88,120]. All five other studies that met our inclusion criteria reported an improvement in academic performance.

Due to heterogeneity in academic assessment methods and high possibility of confounding, we only pooled the studies for which we could extract odds ratio values that: a) measure final year academic scores and b) control for previous academic performance. Three studies met these two inclusion criteria, all of which reported the effect of iBSc degrees. On average, students who took some time off to perform research were twice as likely to outperform their peers (OR = 1.99, 1.39–2.84), even after adjustment for previous academic performance. It is noteworthy that all pooled studies investigated research time-off that was around one year in duration (iBSc), and that the positive effect of research time-off on subsequent academic performance may actually be reversed if the research delays are prolonged. Dyrbye et al pinned down a critical period of three years, after which medical students start to lose clinical knowledge and skills by the time they return to the core medical program [28].

#### IV2. Gender equality in medical student research

There is no apparent gender difference regarding the following outcomes: Interest in a career in research**;** involvement in research during medical school**;** attitudes towards research**;** interest in- or motivation towards- performing research**;** research knowledge or skills. However, on average, males seem to be significantly more likely to publish (or submit for publication) the research they performed during medical school (OR = 1.59, 1.26–2.01). The reasons behind this gender gap in publication are unclear to us, and have been inadequately researched. Since there is no apparent gender difference in research perceptions, attitudes, motivations or knowledge, we suspect that the gender difference in publications is due to factors unrelated to research such as the overall academic environment or psychosocial factors. Indeed, these findings are consistent with a 2006 study by Jagsi et al showing a generalized gender gap in the authorship of academic medical articles in six major medical journals. Whatever the reasons behind gender differences in publication, they underlie a general issue not specific to medical school research [121].

#### IV3. Type and field of research performed by medical students

The majority of medical student research is original in nature (as opposed to literature reviews). We were interested in finding out what percentage of these research projects were in the basic sciences, since this issue is of particular relevance to translational research. We found that the proportion was highly variable between different studies. In four of the five included studies less than half of medical student research was lab-based basic research, and the pooled weighted estimate was 0.32, 0.14–0.49. Given the relevance of research to competitive residency applications, it should not come as a surprise that lab-based projects do not constitute the majority of medical student research. Nonetheless, these results indicate that efforts directed at increasing the number of physician scientists involved in translational research should not only be directed at bolstering research involvement, but also improving the appeal of basic lab-based research to medical students.

#### IV4. Compulsory vs. elective medical school research

The question of whether undergraduate medical research should be made compulsory or elective has been discussed in the literature, and is a matter of debate [37,97,122]. Arguments in favor of mandatory incorporation revolve around the ever-increasing importance of evidence-based clinical practice, while arguments against it revolve around the importance of focusing on clinical skills education. Diez et al. recommended *against* Germany's dissertation requirement, due to the steady decline in the number of successful dissertations [123]. Our results tell a similar story; the fraction of graduation theses resulting in a first-authored higher quality publication was smaller than the overall average (0.07, 0.03–0.14). At first, this may seem counterintuitive, as one may predict that the systematic incorporation of research as a necessary graduation requirement would raise the fraction culminating in a first-authored higher quality publication. However, one needs to bear in mind that since graduation theses are an obligatory requirement, a fraction of those students performing research may not be interested at all in what they are doing. Taking this into consideration, it should not come as a surprise that percentages as high as 34% (Cohen et al [38]) and 31% (Dyrbye et al [29]) of *voluntary* medical student research were reported to result in first-authored Medline-indexed publications. Weihrauch et al and Pabst et al, on the other hand, reported favorable results in terms of the personal and professional value of the German dissertation requirement [124,125].

#### IV5. The situation in countries with developing economies

We retrieved studies that were performed in India [67,72], Uganda [68], China [69], Brazil [70,74], UAE [71], Croatia [73,79], Pakistan [75,77,80,86], Peru [76], and Turkey [78].

The number of medical schools and the research budget in developing countries are alarmingly mismatched with their needs [1]. This disparity, we believe, reflects naturally on the status of medical student research. In fact, medical student research might be even more important in developing countries than in developed countries, due to the pressing need to adapt international standards to local community needs.

Medical students in developing countries arguably face a set of extra challenges and are influenced by a number of different factors in comparison to developed countries [126]. For example, the high student-to-teacher ratio makes it increasingly difficult for medical students to have mentors and role models. Even research based on statistical analysis of patient records is often difficult to perform in many medical schools, due to suboptimal Information and Communications Technology (ICT) infrastructure in hospitals and in teaching premises in countries with developing economies [127]. While excellent research may of course be performed in resource-poor countries, it is preferable that any reform in research funding is coupled with a well-developed educational and managerial infrastructure; otherwise the research output may be largely suboptimal [128]. Worryingly, an essay by Silva et al. reported a decrease in the ratio of Undergraduate Student Research Assistant Programs (USRA's) to the number of undergraduates in Brazil over the past years [129].

Students’ interest in research was higher in countries with developing economies than in developed countries (0.82, 0.67–0.91 vs. 0.47, 0.26–0.69). One possible explanation for this finding is that the lack of opportunities causes higher eagerness to perform research. Another, possibly more likely, explanation is higher career-related anxiety in lower-income settings, with a resultant boost in research interest. Indeed, students in developing countries were not significantly less exposed to research, a result which may be reflective of the higher interest rates, bolstering research engagement despite inadequacies in resources. These results are supported by the findings of Baig et al, showing that 40% of Pakistani medical students viewed research as a tool to secure competitive residencies in the US [86].

## Conclusions and Future Directions

Overall, our review shows that there’s considerable variability in medical student research exposure, engagement and productivity among different medical schools. A large proportion of the medical student population is interested in research, but is deterred by practical difficulties, including the lack of opportunities and funding. The benefits of research exposure on the short- and long-term scientific productivity is well documented in the literature, and a clear correlation is identified between medical school research engagement and later engagement in research projects (including the choice of an academic career). However, the number of well-controlled, high-quality prospective studies on the topic is limited and it is difficult to exclude reverse-causality. Existing evidence suggests that medical school research *does* have a positive effect on the choice of an academic career, but it does so through strengthening pre-existing interest. Financial worries, gender, having a higher degree (MSc or PhD) before matriculation and perceived competitiveness of the residency of choice are among the factors that affect the engagement of medical students in research and their scientific productivity.

Another potential limitation of this review is publication bias. It is conceivable that medical schools where students had a positive experience with research rush to publish their results, whereas others with experiences that were not so positive blamed it on the design of the program without publishing their results. It is also clear that there are plenty of successful undergraduate research programs that do not publish their results.

We suggest that more studies are done to assess the different structural and managerial aspects of standardized undergraduate medical research, as well as the differences between compulsory research components, elective research components, intercalated BSc's and extracurricular research in terms of academic, professional and psycho-cognitive effects. Further, we recommend more investigation into the quality and citation potential of published medical student research in comparison to that of established researchers and physicians.

## Supporting Information

S1 File PRISMA guidelines checklist. (PDF)

S2 File Supplementary methodology file. (PDF)

S3 File Quality assessment and quantitative data extraction sheet. **Abbreviations used: D,** developing countries; **I,** intercalated Bachelor of Science degree (iBSc); **X,** Cross-sectional; **R,** Retrospective; **I**, Interventional; **Pro**, Prospective; **Q,** questionnaire; **DS**, database search; **IN,** interview.
(XLSX)

S4 File Sensitivity plots for the pooled effect size values calculated. (PDF)

S5 File Supplementary tables accompanying the main text. (PDF)

## References

[1] Frenk J, Chen L, Bhutta Z, Cohen J, Crisp N, Evans T, et al Health professionals for a new century: transforming education to strengthen health systems in an interdependent world. Lancet. 2010;376(9756):1923–58. 10.1016/S0140-6736(10)61854-5 21112623

[2] Guelich JM, Singer BH, Castro MC, Rosenberg LE. A gender gap in the next generation of physician-scientists: medical student interest and participation in research. J Investig Med. 2002;50(6):412–8. 12425427 10.1136/jim-50-06-01

[3] Rosenberg LE. Physician-Scientists—Endangered and Essential. Science. 1999;283(5400):331–2. 9925491 10.1126/science.283.5400.331

[4] Mirmira RG. The vulnerable physician-scientist. Mol Endocrinol. 2014;28(5):603–6. 10.1210/me.2014-1085 24786416 PMC4004776

[5] National Institute of General Medical Sciences: Medical Scientist Training Program [Internet]. Available: http://www.nigms.nih.gov/Training/InstPredoc/PredocOverview-MSTP.htm. Accessed 14 April 2015.

[6] Brass LF, Akabas MH, Burnley LD, Engman DM, Wiley CA, Andersen OS. Are MD-PhD programs meeting their goals? An analysis of career choices made by graduates of 24 MD-PhD programs. Acad Med. 2010;85(4):692–701. 10.1097/ACM.0b013e3181d3ca17 20186033 PMC4441397

[7] Association of American Medical Colleges. Medical School Graduation Questionnaire—2013 all school summary report [Internet]. Available: https://www.aamc.org/download/350998/data/2013gqallschoolssummaryreport.pdf. Accessed 14 April 2015.

[8] Fang D, Meyer RE. Effect of two Howard Hughes Medical Institute research training programs for medical students on the likelihood of pursuing research careers. Acad Med. 2003;78(12):1271–80. 14660432 10.1097/00001888-200312000-00017

[9] Gallin EK, Le Blancq S. Launching a New Fellowship for Medical Students : The First Years of the Doris Duke Clinical Research Fellowship Program. J Investig Med. 2005;53(2):73–81. 15810493 10.2310/6650.2005.00202

[10] Langhammer CG, Garg K, Neubauer J, Rosenthal S, Kinzy TG. Medical student research exposure via a series of modular research programs. J Investig Med. 2009;57(1):11–7. 10.231/JIM.0b013e3181946fec 19092679

[11] Zier K, Friedman E, Smith L. Supportive Programs Increase Medical Students’ Research Interest and Productivity. J Investig Med. 2006;54(04):201. 10.2310/6650.2006.05013 17152859

[12] Davis DP, Poste JC, Kelly D. The UCSD Research Associate Program: a recipe for successfully integrating undergraduates with emergency medicine research. J Emerg Med. 2005;28(1):89–93. 15657015 10.1016/j.jemermed.2004.07.012

[13] Rosenblatt R, Desnick L, Corrigan C, Keerbs A. The evolution of a required research program for medical students at the University of Washington School of Medicine. Acad Med. 2006;81(10):877–81. 16985345 10.1097/01.ACM.0000238240.04371.52

[14] Hunter A, Laursen SL, Seymour E. Becoming a scientist: The role of undergraduate research in students’ cognitive, personal, and professional development. Sci Educ. 2007;91(1):36–74.

[15] Lopatto D. Undergraduate research experiences support science career decisions and active learning. CBE Life Sci Educ. 2007;6(4):297–306. 18056301 10.1187/cbe.07-06-0039 PMC2104507

[16] Russell SH, Hancock MP, McCullough J. The pipeline. Benefits of undergraduate research experiences. Science. 2007;316(5824):548–9. 17463273 10.1126/science.1140384

[17] Pacifici LB, Thomson N. Undergraduate science research: a comparison of influences and experiences between premed and non-premed students. CBE Life Sci Educ. 2011;10(2):199–208. 10.1187/cbe.11-01-0005 21633068 PMC3105926

[18] Straus SE, Straus C, Tzanetos K. Career choice in academic medicine: systematic review. J Gen Intern Med. 2006;21(12):1222–9. 17105520 10.1111/j.1525-1497.2006.00599.x PMC1924755

[19] International Monetary Fund. World Economic Outlook Database [Internet]. 2013. Available: http://www.imf.org/external/pubs/ft/weo/2013/01/weodata/index.aspx. Accessed 14 April 2015.

[20] Moher D, Liberati A, Tetzlaff J, Altman DG. Preferred reporting items for systematic reviews and meta-analyses: the PRISMA statement. PLoS Med. 2009;6(7):e1000097 10.1371/journal.pmed.1000097 19621072 PMC2707599

[21] Sandelowski M, Voils CI, Barroso J. Defining and Designing Mixed Research Synthesis Studies. Res Sch. 2006;13(1):29 20098638 PMC2809982

[22] Dixon-woods M, Agarwal S, Young B, Jones D, Sutton A. Integrative approaches to qualitative and quantitative evidence Health Development Agency; 2004 Available: http://citeseerx.ist.psu.edu/viewdoc/download?doi=10.1.1.96.8783&rep=rep1&type=pdf. Accessed 14 April 2015.

[23] Solomon SS, Tom SC, Pichert J, Wasserman D. Impact of medical student research in the development of physician-scientists. J Investig Med. 2003;51(3):149–56. 12769197 10.1136/jim-51-03-17

[24] Nieminen P, Sipilä K, Takkinen H, Renko M, Risteli L. Medical theses as part of the scientific training in basic medical and dental education: experiences from Finland. BMC Med Educ. 2007;7:51 18053247 10.1186/1472-6920-7-51 PMC2235851

[25] Chusid MJ, Havens PL, Coleman CN. Alpha omega alpha election and medical school thesis publication: relationship to subsequent publication rate over a twenty-year period. Yale J Biol Med. 1993;66(2):67–73. 8303911 PMC2588837

[26] Segal S, Lloyd T, Houts PS, Stillman PL, Jungas RL, Greer RB. The association between students’ research involvement in medical school and their postgraduate medical activities. Acad Med. 1990;65(8):530–3. 2383337 10.1097/00001888-199008000-00010

[27] Salmi LR, Gana S, Mouillet E. Publication pattern of medical theses, France, 1993–98. Med Educ. 2001;35(1):18–21. 11123590 10.1046/j.1365-2923.2001.00768.x

[28] Dyrbye LN, Thomas MR, Natt N, Rohren CH. Prolonged delays for research training in medical school are associated with poorer subsequent clinical knowledge. J Gen Intern Med. 2007;22(8):1101–6. 17492473 10.1007/s11606-007-0200-x PMC2305740

[29] Dyrbye LN, Davidson LW, Cook DA. Publications and presentations resulting from required research by students at Mayo Medical School, 1976–2003. Acad Med. 2008;83(6):604–10. 10.1097/ACM.0b013e3181723108 18520471

[30] Cursiefen C, Altunbas A. Contribution of medical student research to the Medline-indexed publications of a German medical faculty. Med Educ. 1998;32(4):439–40. 9743810 10.1046/j.1365-2923.1998.00255.x

[31] Smith WH, Rogers JG, Hansen TN, Smith C V. Early Career Development in Academic Pediatrics of Participants in the APS-SPR Medical Student Research Program. Pediatr Res. 2009;65(4):474–7. 10.1203/PDR.0b013e3181975f85 19092716 PMC2761208

[32] Brancati FL, Mead L, Levine DM, Martin D, Margolis S, Klag MJ. Early predictors of career achievement in academic medicine. JAMA. 1992;267(10):1372–6. 1740860

[33] Hunskaar S, Breivik J, Siebke M, Tømmerås K, Figenschau K, Hansen J. Evaluation of the medical student research programme in Norwegian medical schools. A survey of students and supervisors. BMC Med Educ. 2009;9:43 10.1186/1472-6920-9-43 19602226 PMC2720957

[34] Reinders JJ, Kropmans TJB, Cohen-Schotanus J. Extracurricular research experience of medical students and their scientific output after graduation. Med Educ. 2005;39(2):237 15679693 10.1111/j.1365-2929.2004.02078.x

[35] Jacobs CD, Cross PC. The value of medical student research: the experience at Stanford University School of Medicine. Med Educ. 1995;29(5):342–6. 8699971 10.1111/j.1365-2923.1995.tb00023.x

[36] Dorrance K, Denton GD, Proemba J, La Rochelle J, Nasir J, Argyros G, et al An internal medicine interest group research program can improve scholarly productivity of medical students and foster mentoring relationships with internists. Teach Learn Med. 2008;20(2):163–7. 10.1080/10401330801991857 18444204

[37] Frishman WH. Student Research Projects and Theses. Hear Dis. 2001;3(3):140–4. 10.1097/00132580-200105000-00002 11975783

[38] Cohen BL, Friedman E, Zier K. Publications by students doing a year of full-time research: what are realistic expectations? Am J Med. 2008;121(6):545–8. 10.1016/j.amjmed.2008.03.006 18501238

[39] Zorzi A, Rourke J, Kennard M, Peterson ME, Miller KJ. Combined research and clinical learning make rural summer studentship program a successful model. Educ Health (Abingdon). 2005;18(3):329–37. 16236581 10.1080/13576280500289330

[40] Wagne RF, Wagner KD. Senior Medical Student Clinical and Research Electives in Dermatologic Surgery. Int J Dermatol. 1992;31(4):288–90. 1634298 10.1111/j.1365-4362.1992.tb03576.x

[41] Van Eyk HJ, Hooiveld MHW, Van Leeuwen TN, Van der Wurff BLJ, De Craen AJM, Dekker FW. Scientific output of Dutch medical students. Med Teach. 2010;32(3):231–5. 10.3109/01421591003596592 20218838

[42] Houlden RL, Raja JB, Collier CP, Clark AF, Waugh JM. Medical students’ perceptions of an undergraduate research elective. Med Teach. 2004;26(7):659–61. 15763861 10.1080/01421590400019542

[43] Chongsiriwatana K, Phelan S, Skipper B, Rhyne R, Rayburn W. Required research by medical students and their choice of a women ‘ s health care residency. Am J Obstet Gynecol. 2005;192(5):1478–80. 15902143 10.1016/j.ajog.2005.01.011

[44] Halder N, Hadjidemetriou C, Pearson R, Farooq K, Lydall GJ, Malik A, et al Student career choice in psychiatry: findings from 18 UK medical schools. Int Rev Psychiatry. 2013;25(4):438–44. 10.3109/09540261.2013.824414 24032499

[45] Sánchez JP, Peters L, Lee-Rey E, Strelnick H, Garrison G, Zhang K, et al Racial and ethnic minority medical students’ perceptions of and interest in careers in academic medicine. Acad Med. 2013;88(9):1299–307. 10.1097/ACM.0b013e31829f87a7 23887018

[46] Riley SC, Morton J, Ray DC, Swann DG, Davidson DJ. An integrated model for developing research skills in an undergraduate medical curriculum: appraisal of an approach using student selected components. Perspect Med Educ. 2013;2(4):230–47. 10.1007/s40037-013-0079-7 24037741 PMC3792228

[47] Burgoyne LN, O’Flynn S, Boylan GB. Undergraduate medical research: the student perspective. Med Educ Online. 2010;15. 10.3402/meo.v15i0.5212 PMC2939395 20844608

[48] Griffin MF, Hindocha S. Publication practices of medical students at British medical schools: experience, attitudes and barriers to publish. Med Teach. 2011;33(1):e1–8. 10.3109/0142159X.2011.530320 21182368

[49] Remes V, Helenius I, Sinisaari I. Research and medical students. Med Teach. 2000;22(2):164–7.

[50] Dewey M. Students’ evaluation of research during medical studies: medical dissertation in Germany. Med Educ. 2003;37(3):278–278. 12603767 10.1046/j.1365-2923.2003.14581.x

[51] Lloyd T, Phillips BR, Aber RC. Factors that influence doctors’ participation in clinical research. Med Educ. 2004;38(8):848–51. 15271045 10.1111/j.1365-2929.2004.01895.x

[52] Siemens DR, Punnen S, Wong J, Kanji N. A survey on the attitudes towards research in medical school. BMC Med Educ. 2010;10:4 10.1186/1472-6920-10-4 20096112 PMC2823602

[53] Pheley A, Lois H, J S. Interests in research electives among osteopathis medical students. J Am Osteopath Assoc. 2006;106(11):667–70. 17192455

[54] Cruser DA, Dubin B, Brown SK, Bakken LL, Licciardone JC, Podawiltz AL, et al Biomedical research competencies for osteopathic medical students. Osteopath Med Prim Care. 2009;3:10 10.1186/1750-4732-3-10 19825171 PMC2770523

[55] Galletly C, Chur-Hansen A, Air T, Chapman I. Academics of the future? A survey of final year medical students. Australas Psychiatry. 2009;17(6):502–5. 20001376 10.1080/10398560903284935

[56] Kim K, Park J, Lee Y, Choi K. What is different about medical students interested in non-clinical careers? BMC Med Educ. 2013;13:81 10.1186/1472-6920-13-81 23731551 PMC3679731

[57] Yamazaki Y, Uka T, Shimizu H, Miyahira A, Sakai T, Marui E. Japanese medical students’ interest in basic sciences: a questionnaire survey of a medical school in Japan. Tohoku J Exp Med. 2013;229(2):129–36. 23337622 10.1620/tjem.229.129

[58] Greenberg RB, Ziegler CH, Borges NJ, Elam CL, Stratton TD, Woods S. Medical student interest in academic medical careers: a multi-institutional study. Perspect Med Educ. 2013; 2(5–6):298–316. 10.1007/s40037-013-0074-z 23670688 PMC3824757

[59] Nicholson J, Cleland J, Lemon J, Galley HF. Why medical students choose not to carry out an intercalated BSc: a questionnaire study. BMC Med Educ. 2010;10:25 10.1186/1472-6920-10-25 20331878 PMC2850914

[60] Tait N, Marshall T. Is an intercalated BSc degree associated with higher marks in examinations during the clinical years? Med Educ. 1995;29(3):216–9. 7623715 10.1111/j.1365-2923.1995.tb02833.x

[61] Park SJ, Liang MM, Sherwin TT, McGhee CN. Completing an intercalated research degree during medical undergraduate training: barriers, benefits and postgraduate career profiles. N Z Med J. 2010;123(1323):24–33. 20930907

[62] Mahesan N, Crichton S, Sewell H, Howell S. The effect of an intercalated BSc on subsequent academic performance. BMC Med Educ. 2011;11(1):76. 21967682 10.1186/1472-6920-11-76 PMC3200165

[63] Cleland J a, Milne A, Sinclair H, Lee AJ. An intercalated BSc degree is associated with higher marks in subsequent medical school examinations. BMC Med Educ. 2009;9:24 10.1186/1472-6920-9-24 19454007 PMC2689211

[64] Collins JP, Farish S, McCalman JS, McColl GJ. A mandatory intercalated degree programme: revitalising and enhancing academic and evidence-based medicine. Med Teach. 2010;32(12):e541–6. 10.3109/0142159X.2010.528807 21090941

[65] Nguyen-Van-Tam JS, Logan RF, Logan S, Mindell JS. What happens to medical students who complete an honours year in public health and epidemiology? Med Educ. 2001;35(2):134–6. 11169085 10.1046/j.1365-2923.2001.00774.x

[66] Evered DC, Anderson J, Griggs P, Wakeford R. The correlates of research success. Br Med J (Clin Res Ed). 1987;25;295(6592):241–6. 10.1136/bmj.295.6592.241 PMC1247081 3115391

[67] Harsha Kumar H, Jayaram S, Kumar GS, Vinita J, Rohit S, Satish M, et al Perception, Practices Towards Research and Predictors of Research Career Among UG Medical Students from Coastal South India: A Cross-Sectional Study. Indian J Community Med. 2009;34(4):306–9. 10.4103/0970-0218.58388 20165623 PMC2822190

[68] Munabi IG, Katabira ET, Konde-Lule J. Early undergraduate research experience at Makerere University Faculty of Medicine : a tool for promoting medical research. Afr Health Sci. 2006;6(3):182–6. 17140343 10.5555/afhs.2006.6.3.182 PMC1831889

[69] She L, Wu B, Xu L, Wu J, Zhang P, Li E. Determinants of career aspirations of medical students in southern China. BMC Med Educ. 2008;8:59 10.1186/1472-6920-8-59 19077214 PMC2621218

[70] Fede AB, Miranda MDC, Lera AT, Ueda A, Antonangelo DV, Schaffhausser HDL, et al Experience with the ABC Foundation School of Medicine undergraduate meeting. Rev Assoc Med Bras. 2010;56(3):313–7. 20676539 10.1590/s0104-42302010000300016

[71] Mclean M, Howarth FC. Does Undergraduate Student Research Constitute Scholarship ? Drawing on the Experiences of One Medical Faculty. J Scholarship Teach Learn. 2008;8(1):72–87.

[72] Mitra S, Goyal S, Muliyil JP, Jacob K. Attitude, concerns and conduct of research among medical students. Natl Med J India. 2006;19(6):345–7. 17343024

[73] Kolcić I, Polasek O, Mihalj H, Gombac E, Kraljević V, Kraljević I, et al Research involvement, specialty choice, and emigration preferences of final year medical students in croatia. Croat Med J. 2005 2;46(1):88–95. 15726681

[74] de Oliveira NA, Luz MR, Saraiva RM, Alves LA. Student views of research training programmes in medical schools. Med Educ. 2011;45(7):748–55. 10.1111/j.1365-2923.2011.03986.x 21649708

[75] Ejaz K, Shamim M, Hussain S. Involvement of medical students and fresh medical graduates of Karachi, Pakistan in research. J Pak Med Assoc. 2011;61(2):115–20. 21375155

[76] Arriola-Quiroz I, Curioso WH, Cruz-Encarnacion M, Gayoso O. Characteristics and publication patterns of theses from a Peruvian medical school. Health Info Libr J. 2010;27(2):148–54. 10.1111/j.1471-1842.2010.00878.x 20565556

[77] Khan H, Taqui AM, Khawaja MR, Fatmi Z. Problem-based versus conventional curricula: influence on knowledge and attitudes of medical students towards health research. PLoS One. 2007;2(7):e632 17637847 10.1371/journal.pone.0000632 PMC1913552

[78] Akman M, Unalan PC, Kalaca S, Kaya CA, Cifcili S, Uzuner A. A three-year mandatory student research program in an undergraduate medical curriculum in Turkey. Kuwait Med J. 2012;42(3):205–10.

[79] Hren D, Lukić IK, Marusić A, Vodopivec I, Vujaklija A, Hrabak M, et al Teaching research methodology in medical schools: students’ attitudes towards and knowledge about science. Med Educ. 2004;38(1):81–6. 14962029 10.1111/j.1365-2923.2004.01735.x

[80] Khan H, Khawaja MR, Waheed A, Rauf MA, Fatmi Z. Knowledge and attitudes about health research amongst a group of Pakistani medical students. BMC Med Educ. 2006;6:54 17081286 10.1186/1472-6920-6-54 PMC1635552

[81] Gerrard JM, Fish I, Tate R, Fish DG. Evaluation of the careers of graduates of the University of Manitoba’s BSc (Medicine) program. CMAJ. 1988;139(11):1063–8. 3191444 PMC1268442

[82] Stubbs TA, Lightman EG, Mathieson P. Is it intelligent to intercalate? A two centre cross-sectional study exploring the value of intercalated degrees, and the possible effects of the recent tuition fee rise in England. BMJ Open. 2013;3(1). 10.1136/bmjopen-2012-002193 PMC3563132 23355672

[83] Wyllie AH, Currie AR. The Edinburgh intercalated honours BSc in pathology: evaluation of selection methods, undergraduate performance, and postgraduate career. Br Med J (Clin Res Ed). 1986;292(6536):1646–8. 3087558 10.1136/bmj.292.6536.1646 PMC1340712

[84] MacGowan Alastair P., Johnston Peter W. and AWT. Intercalated degrees. Br Med J (Clin Res Ed). 1986;293(6540):201. 10.1136/bmj.293.6540.201-a PMC1340925 3089447

[85] McManus IC, Richards P, Winder BC. Intercalated degrees, learning styles, and career preferences: prospective longitudinal study of UK medical students. BMJ. 1999;319(7209):542–6. 10463892 10.1136/bmj.319.7209.542 PMC28204

[86] Baig SA, Hasan SA, Ahmed SM, Ejaz K, Aziz S, Dohadhwala NA. Reasons behind the increase in research activities among medical students of Karachi, Pakistan, a low-income country. Educ Health (Abingdon).2013;26(2):117–21. 10.4103/1357-6283.120705 24200734

[87] Williamson JD. Intercalated degrees. Br Med J (Clin Res Ed). 1986;293(6542):336.

[88] Howman M, Jones M. Does undertaking an intercalated BSc influence first clinical year exam results at a London medical school? BMC Med Educ. 2011;11:6 10.1186/1472-6920-11-6 21291522 PMC3053587

[89] Eaton DG, Thong YH. The Bachelor of Medical Science research degree as a start for clinician-scientists. Med Educ. 1985;19(6):445–51. 4069022 10.1111/j.1365-2923.1985.tb01352.x

[90] Murdoch-Eaton D, Drewery S, Elton S, Emmerson C, Marshall M, Smith J a, et al What do medical students understand by research and research skills? Identifying research opportunities within undergraduate projects. Med Teach. 2010;32(3):e152–60. 10.3109/01421591003657493 20218832

[91] Jacobs CD, Cross PC. The value of medical student research: the experience at Stanford University School of Medicine. Med Educ. 1995;29(5):342–6. 8699971 10.1111/j.1365-2923.1995.tb00023.x

[92] Houlden RL, Raja JB, Collier CP, Clark AF, Waugh JM. Medical students’ perceptions of an undergraduate research elective. Med Teach. 2004;26(7):659–61. 15763861 10.1080/01421590400019542

[93] Reinders JJ, Kropmans TJB, Cohen-Schotanus J. Extracurricular research experience of medical students and their scientific output after graduation. Med Educ. 2005;39(2):237 15679693 10.1111/j.1365-2929.2004.02078.x

[94] Forrest JN. The medical student thesis at Yale. Yale J Biol Med. 1989;62(3):291–2. 2815841 PMC2589109

[95] Laskowitz DT, Drucker RP, Parsonnet J, Cross PC, Gesundheit N. Engaging students in dedicated research and scholarship during medical school: the long-term experiences at Duke and Stanford. Acad Med. 2010;85(3):419–28. 10.1097/ACM.0b013e3181ccc77a 20182114

[96] Boninger M, Troen P, Green E, Borkan J, Lance-Jones C, Humphrey A, et al Implementation of a longitudinal mentored scholarly project: an approach at two medical schools. Acad Med. 2010;85(3):429–37. 10.1097/ACM.0b013e3181ccc96f 20182115

[97] Parsonnet J, Gruppuso P a, Kanter SL, Boninger M. Required vs. elective research and in-depth scholarship programs in the medical student curriculum. Acad Med. 2010;85(3):405–8. 10.1097/ACM.0b013e3181cccdc4 20182112

[98] Solomon SS, Tom SC, Pichert J, Wasserman D, Powers AC. Impact of medical student research in the development of physician-scientists. J Investig Med. 2003;51(3):149–56. 12769197 10.1136/jim-51-03-17

[99] Jones M, Singh S, Lloyd M. “It isn’t just consultants that need a BSc”: student experiences of an Intercalated BSc in primary health care. Med Teach. 2005;27(2):164–8. 16019339 10.1080/01421590400019567

[100] O’Sullivan PS, Niehaus B, Lockspeiser TM, Irby DM. Becoming an academic doctor: perceptions of scholarly careers. Med Educ. 2009;43(4):335–41. 10.1111/j.1365-2923.2008.03270.x 19335575

[101] Shapiro J, Coggan P, Rubel a, Morohasi D, Fitzpatrick C, Danque F. The process of faculty-mentored student research in family medicine: motives and lessons. Fam Med. 1994;26(5):283–9. 8050645

[102] Reynolds HY. In choosing a research health career, mentoring is essential. Lung. 2008;186:1–6. 17990035 10.1007/s00408-007-9050-x

[103] Robinson L, Drewery S, Ellershaw J, Smith J, Whittle S, Murdoch-Eaton D. Research governance: impeding both research and teaching? A survey of impact on undergraduate research opportunities. Med Educ. 2007;41(8):729–36. 17661880 10.1111/j.1365-2923.2007.02776.x

[104] Mcgee R, Keller JL. Identifying Future Scientists : Predicting Persistence into Research Training. CBE Life Sci Educ. 2007;6:316–31. 18056303 10.1187/cbe.07-04-0020 PMC2104502

[105] Lawson McLean A, Saunders C, Velu PP, Iredale J, Hor K, Russell CD. Twelve tips for teachers to encourage student engagement in academic medicine. Med Teach. 2013;35(7):549–54. 10.3109/0142159X.2013.775412 23496123

[106] Dorrance K, Denton GD, Proemba J, La Rochelle J, Nasir J, Argyros G, et al An internal medicine interest group research program can improve scholarly productivity of medical students and foster mentoring relationships with internists. Teach Learn Med. 2008;20(2):163–7. 10.1080/10401330801991857 18444204

[107] Journal of Young Investigators [Internet]. Available: http://www.jyi.org/site/. Accessed 14 April 2015.

[108] Internationl Medical Journal of Students’ Research [Internet]. Available: http://www.imjsr.org/. Accessed 14 April 2015.

[109] McGill Journal of Medicine [Internet]. Available: http://www.med.mcgill.ca/mjm/. Accessed 14 April 2015.

[110] American Journal of Undergraduate Research [Internet]. Available: http://www.ajur.uni.edu/index.html. Accessed 14 April 2015.

[111] Yale University School of Medicine: 2007 student thesis abstracts. Yale J Biol Med. 2007;80(1):1–38. 18170929 PMC2048992

[112] Weber EJ, Callaham ML, Wears RL, Barton C, Young G. Unpublished research from a medical specialty meeting: why investigators fail to publish. JAMA. 1998;280(3):257–9. 9676674 10.1001/jama.280.3.257

[113] Riveros C, Dechartres A, Perrodeau E, Haneef R, Boutron I, Ravaud P. Timing and completeness of trial results posted at ClinicalTrials.gov and published in journals. PLoS Med. 2013;10(12):e1001566 10.1371/journal.pmed.1001566 24311990 PMC3849189

[114] O’Sullivan PS, Niehaus B, Lockspeiser TM, Irby DM. Becoming an academic doctor: perceptions of scholarly careers. Med Educ. 2009;43(4):335–41. 10.1111/j.1365-2923.2008.03270.x 19335575

[115] Leow JJ, Mackay SD, Grigg MJ, Haider AH. Surgical research elective in the United States: an Australian medical student’s experience. J Surg Educ. 2011;68(6):562–7. 10.1016/j.jsurg.2011.06.002 22000544

[116] Agha R, Singh G. Letters to the editor: Studying for an intercalated BSc. Med Educ. 2003;37:839 12950949 10.1046/j.1365-2923.2003.01604.x

[117] Aslam F, Shakir M, Qayyum MA. Why medical students are crucial to the future of research in South Asia. PLoS Med. 2005;2(11):e322 16288553 10.1371/journal.pmed.0020322 PMC1297528

[118] Simunovic F. Is there a place for medical students in research laboratories? A student’s perspective. Med Teach. 2008;30(9–10):875–6. 10.1080/01421590802314889 18825562

[119] Jones M, Hutt P, Eastwood S, Singh S. Impact of an intercalated BSc on medical student performance and careers: a BEME systematic review: BEME Guide No. 28. Med Teach. 2013;35(10):e1493–510. 10.3109/0142159X.2013.806983 23962229

[120] Tait N, Marshall T. Is an intercalated BSc degree associated with higher marks in examinations during the clinical years? Med Educ. 1995 5;29(3):216–9. 7623715 10.1111/j.1365-2923.1995.tb02833.x

[121] Jagsi R, Guancial EA, Worobey CC, Henault LE, Chang Y, Starr R, et al The “gender gap” in authorship of academic medical literature—a 35-year perspective. N Engl J Med. 2006;355(3):281–7. 16855268 10.1056/NEJMsa053910

[122] Ogunyemi D, Bazargan M, Norris K, Jones-quaidoo S, Wolf K, Edelstein R, et al The Development of A Mandatory Medical Thesis in an Urban Medical School. Teach Learn Med. 2005;17(4):363–9. 16197324 10.1207/s15328015tlm1704_9

[123] Diez C, Arkenau CD, Meyer-Wentrup FD. The German Medical Dissertation-Time to Change ? Acad Med. 2000;75(8):861–3. 10965870 10.1097/00001888-200008000-00024

[124] Weihrauch M, Weber A, Pabst R, Weltle D, Lehnert G. The medical dissertation. An assessment from the viewpoint of successful and unsuccessful candidates. Medizinische Klin. 2000;95(10):545–7. 11092166 10.1007/pl00002060

[125] Pabst R, Park D, Paulmann V. The German academic degree “Dr. med.” is better than its reputation. Results of a questionnaire of doctoral students. Dtsch medizinische Wochenschrift. 2012;137(45):2311–5. 10.1055/s-0032-1327241 23111793

[126] Puertas EB, Arósquipa C, Gutiérrez D. Factors that influence a career choice in primary care among medical students from high-, middle-, and low-income countries: a systematic review. Rev Panam Salud Publica. 2013;34(5):351–8. 24553763

[127] Williams CD, Pitchforth EL, O’Callaghan C. Computers, the Internet and medical education in Africa. Med Educ. 2010;44(5):485–8. 10.1111/j.1365-2923.2009.03602.x 20518986

[128] Saldaña-Gastulo JJ, Quezada-Osoria CC, Peña-Oscuvilca A, Mayta-Tristán P. High frequency of plagiarism in medical thesis from a Peruvian public university. Rev Peru Med Exp Salud Publica. 2010;27(1):63–7. 21072452 10.1590/s1726-46342010000100011

[129] Silva T, da Cunha Aguiar LC, Leta J, Santos DO, Cardoso FS, Cabral LM, et al Role of the undergraduate student research assistant in the new millennium. Cell Biol Educ. 2004;3(4):235–40. 15592596 10.1187/cbe.04-02-0032 PMC533125

