# Supplementary material for: Medical Student Research: An Integrated Mixed-Methods Systematic Review and Meta-Analysis
Source: PLoS One. 2015 Jun 18;10(6):e0127470. doi: 10.1371/journal.pone.0127470 (PMC4472353; doi:10.1371/journal.pone.0127470)
Supplement: S2 File — (PDF) [file pone.0127470.s002.pdf]

SUPPLEMENTARY METHODOLOGY

Medical Student Research: An Integrated Mixed-Methods Systematic Review and Metaanalysis

Mohamed Amgad[1 2], Marco Man Kin Tsui[2], Sarah J. Liptrott[3] and Emad Shash[4 5]

1. *Faculty of Medicine, Cairo University, Cairo, Egypt*
2. *Okinawa Institute of Science and Technology Graduate University, Japan*
3. *European Institute of Oncology (IEO), Milano, Italy*
4. *National Cancer Institute, Cairo University, Egypt*
5. *European Organization For Research & Treatment of Cancer (EORTC) Headquarters, Brussels, Belgium*

In this supplementary material, we will describe further details about our mixed-methods systematic review methodology. Citation screening, study selection and quantitative-quantitative integration methods were already described in the main text. This supplementary methodology file describes three aspects of our methodology: (i) outcome-specific quality assessment, (ii) statistical methods used, and (iii) strategy used to handle heterogeneity and publication bias.

a. Outcome-specific quality assessment:

Quality was assessed on an outcome-by-outcome basis (rather than a study-by-study basis) for inclusion into the meta-analysis. That is, the suitability of the study design for each outcome determined its inclusion into-, or exclusion from- the meta-analysis. The data extraction sheet, along with the outcome-specific quality assessment, was implemented in Microsoft Excel and is available as **Supplementary File 2**.

Outcomes were excluded from the meta-analysis if they satisfied any of the following criteria:

i. The study methodology is not suitable for measuring the outcome. A set or pre-specified quality criteria was used for this purpose.

ii. The outcome cannot be grouped with other outcomes in the same category due to: a) *Ambiguous wording:* for example, some studies mention the proportion of medical student research that resulted in "a publication" or "a manuscript" without explicitly mentioning that the publication was a peer-reviewed journal publication. b) *Different sub-category*, or *c) Alternative outcome reporting:* for example, some studies reported mean and standard deviation of knowledge tests without providing a way for their data to be dichotomized for odds ratio calculation. As mentioned earlier, we tried to minimize this loss by contacting the authors of these studies whenever possible to obtain the raw data.

We used the following quality criteria to assess the suitability of study design for each outcome:

i. *Study type:* Types include cross-sectional, retrospective and prospective/interventional design. We assessed the type on an outcome-specific basis. For example, some studies looked at the cross-sectional distribution of a particular outcome, but examined the effect of past exposure of another outcome on a retrospective basis.
ii. *Study design:* Whether the study relied on self-reported questionnaires, interviews or database searching.
iii. *Presence of a control (untreated) group:* This is only relevant for interventional or associative outcomes.
iv. *Control for confounding factors:* Whenever relevant.
v. *Number of institutions at which the study was performed:* This carried relatively higher importance for explorative outcomes (eg. proportion of medical student research that results in a peer-reviewed publication) than associative or interventional outcomes.
vi. *Presence of other limitations:* Of particular relevance is the response rate to questionnaire-based studies. We considered 60% to be the threshold value needed to guard against responder bias.

For each outcome measure, studies of high or medium quality were admitted into the meta-analysis, and studies of low quality were excluded. Similar outcomes that have been admitted into the meta-analysis were grouped together for pooled effect size calculation. Oftentimes a single study reported two or more outcomes that essentially reflect the same outcome measure; only one of those outcomes was used for the meta-analysis. Studies performed in developing countries and studies assessing the effect of an iBSc degree were marked in the figures and, where relevant, pooled to calculate a stratified effect size.

b. Statistical methods used:

Whenever available, raw numbers were reported alongside the percentages or odds ratios. Where studies failed to report the raw number of participants supporting an outcome measure, the raw numbers were inferred using the following method: a) Multiplying the reported percentage (which is often an approximate value) with the total value, b) Rounding to the nearest whole number, c) The exact percentage (and confidence interval, where relevant) was then calculated from the reported total and inferred raw number.

Unadjusted odds ratios were calculated using the equation:

$$OR = \frac{P_E / N_E}{P_{NE} / N_{NE}}$$

where P_E are positive cases (supporting the outcome measure) among the exposed group, N_E are negative cases among the exposed group, P_{NE} are positive cases among the non-exposed group and N_{NE} are negative cases among the non-exposed group. We made every effort to use adjusted odds ratios for the analysis; which have been controlled for various confounding factors. We used the adjusted values were they reported in the included studies, or obtained the adjusted values ourselves, from raw data received directly from the authors, using binary logistic regression in IBM SPSS Version 19.0 [1]. Dichotomization of outcomes was done in a context-dependent manner, but was most often done around the median value to minimize the distorting effect of outliers.

We used the “metafor” package in R to perform the meta-analysis [2]. Odds ratios were log transformed to calculate pooled effect sizes, and after the calculations were performed the results were back-transformed for easier interpretation. Normality of outcomes was assessed using Shapiro-Wilk test as well as visual inspection

of Q-Q Plots. Mean pooled effect size was calculated using a random effects model, to account for the heterogeneity of outcome measures and study settings. Where proportions were not found to be normal, the following was done (using the same methodology as Daniele Fanelli [3]):

i. The proportions were logit-transformed using the following equation for effect size:

$$ES = Log_e\left[\frac{p}{(1-p)}\right]$$

Where *p* is the proportion of respondents supporting the outcome measure being examined.

ii. Normality was assessed. If the logit-transformed values were still not normal, we did not pool the data, and simply showed the raw numbers and percentages in tables. If, on the other hand, the data was normal, we carried on with the mean pooled effect size calculation.

iii. The standard errors (SE) and weights (W) were calculated using the following equations:

$$SE = \sqrt{\left(\frac{1}{np} + \frac{1}{n(1-p)}\right)}$$

$$W = \frac{1}{SE^2} = np(1-p)$$

Where *n* is the total number of respondents.

iv. The values and pooled outcomes were back-transformed to facilitate their interpretation using the following equation:

$$p = \frac{e^x}{e^x + 1}$$

Where x is either the effect size or each of the corresponding 95% confidence interval values.

c. A note on study heterogeneity and publication bias:

Cochrane's Q test was used for assessing heterogeneity. However, despite applying what we believe is a strict outcome admission criteria into the meta-analysis, many of the outcomes were very heterogeneous. There could be many reasons behind this heterogeneity including:

i. *Heterogeneity of study subjects:* Medical students in different countries are selected using different admission criteria and are subjected to different educational curricula.

ii. *Heterogeneity of methodology:* Different studies use different scales to assess the academic performance of medical students or their attitudes towards research.

iii. *Heterogeneity of study settings:* Medical schools within the same country can vary substantially in their curricula and research funding (not to mention medical schools in different countries). This introduces heterogeneity, which is particularly relevant for explorative outcomes (eg. proportion of medical student research that results in a publication, or percentage of medical students exposed to non-mandatory research).

We followed the guidelines presented in the Cochrane Handbook for Systematic Reviews and Interventions in handling the heterogeneity in our data [4]. Hence we used the following strategy:

i. We used a random effects model for calculation of pooled effect sizes.

ii. We avoided effect size calculation altogether for some outcomes where heterogeneity was judged to be caused by incompatibility of grouped outcomes rather than true heterogeneity of effects.

iii. Beside the three potential reasons mentioned earlier, we presented outcome-specific potential reasons of heterogeneity amid the discussion of our results.

iv. We calculated stratified effect sizes of subgroups whenever appropriate.

v. We provided sensitivity analysis plots for the included forest plots (**Supplementary File 3**). Removing one study at a time and calculating the resultant effect size generated the sensitivity plots. Any significant changes to the pooled effect sizes were discussed.

Due to the relatively small number of studies included in the effect size calculation of many variables, and the high heterogeneity observed, we did not use funnel plots to assess for publication bias [5]. Instead, we assessed the robustness of our conclusions using subgroup analysis and sensitivity analysis. The results of these analyses were used to address any potential sources of bias and assess the overall strength of conclusions in a context-dependent manner.

References

1. IBM SPSS Statistics for Windows, Version 19.0. Armonk, NY: IBM Corp. (2010).

2. Viechtbauer W (2010) Conducting meta-analyses in R with the metafor package. J Stat Softw 36: 1–48. Available: http://www.jstatsoft.org/v36/i03/.

3. Fanelli D (2009) How many scientists fabricate and falsify research? A systematic review and meta-analysis of survey data. PLoS One 4: e5738. Available: http://www.pubmedcentral.nih.gov/articlerender.fcgi?artid=2685008&tool=pmcentrez&rendertype=abstract. Accessed 18 July 2014.

4. Collaboration TC (2009) Cochrane Handbook for Systematic Reviews of Interventions Version 5.0.2 [updated September 2009]. JPT H, Green S, editors. Available: www.cochrane-handbook.org.

5. Terrin N, Schmid CH, Lau J, Olkin I (2003) Adjusting for publication bias in the presence of heterogeneity. Stat Med 22: 2113–2126. Available: http://www.ncbi.nlm.nih.gov/pubmed/12820277. Accessed 17 August 2014.
