# Supplementary material for: Medical Student Research: An Integrated Mixed-Methods Systematic Review and Meta-Analysis
Source: PLoS One. 2015 Jun 18;10(6):e0127470. doi: 10.1371/journal.pone.0127470 (PMC4472353; doi:10.1371/journal.pone.0127470)

I.1.a. Interest in performing research (All) :

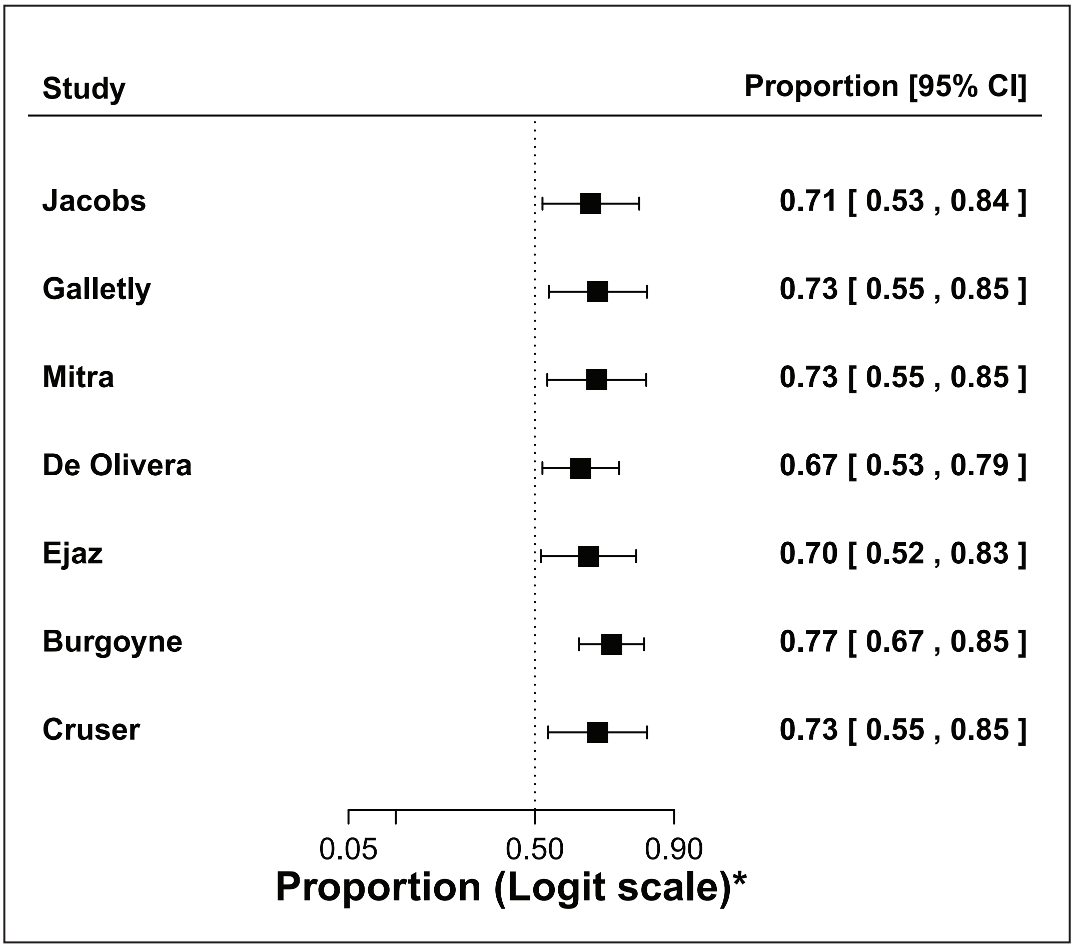

I.1.b. Interest in a career involving research (All) :

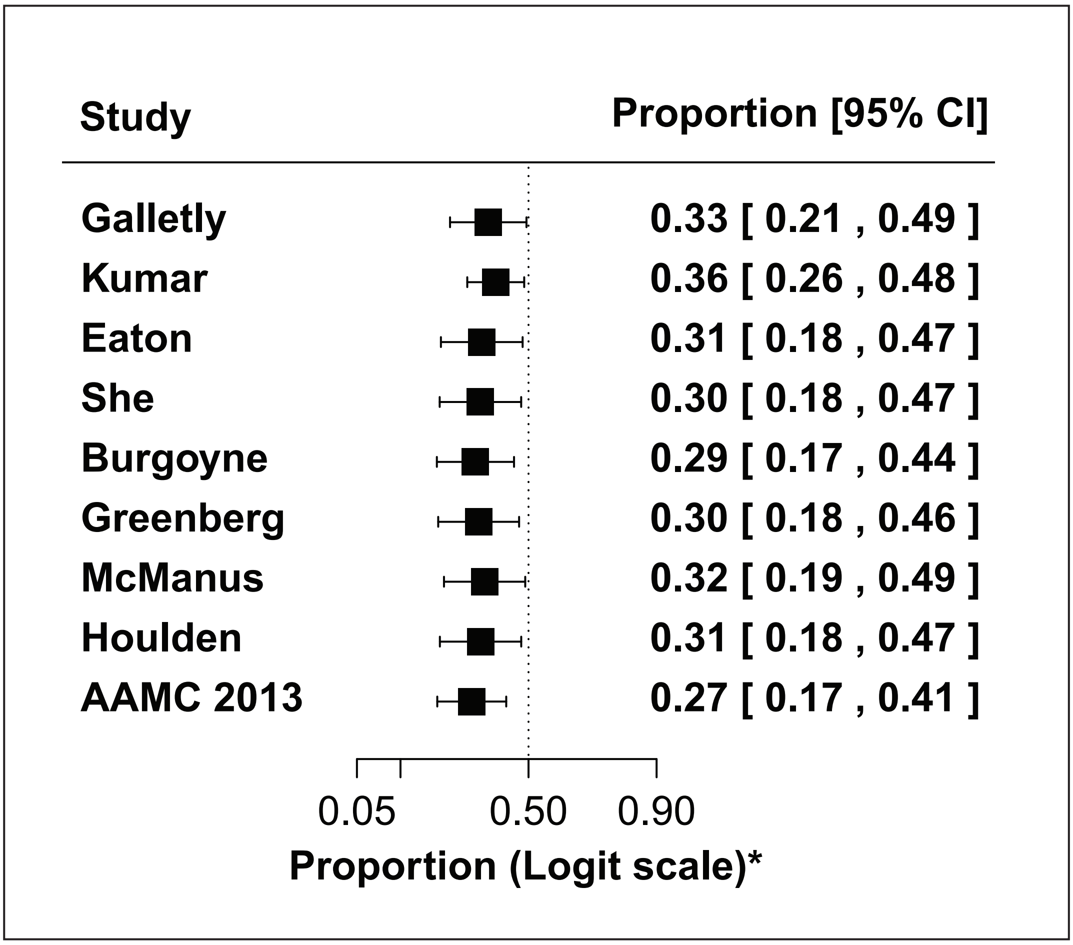

I.2. Exposure of medical students to research (All) :

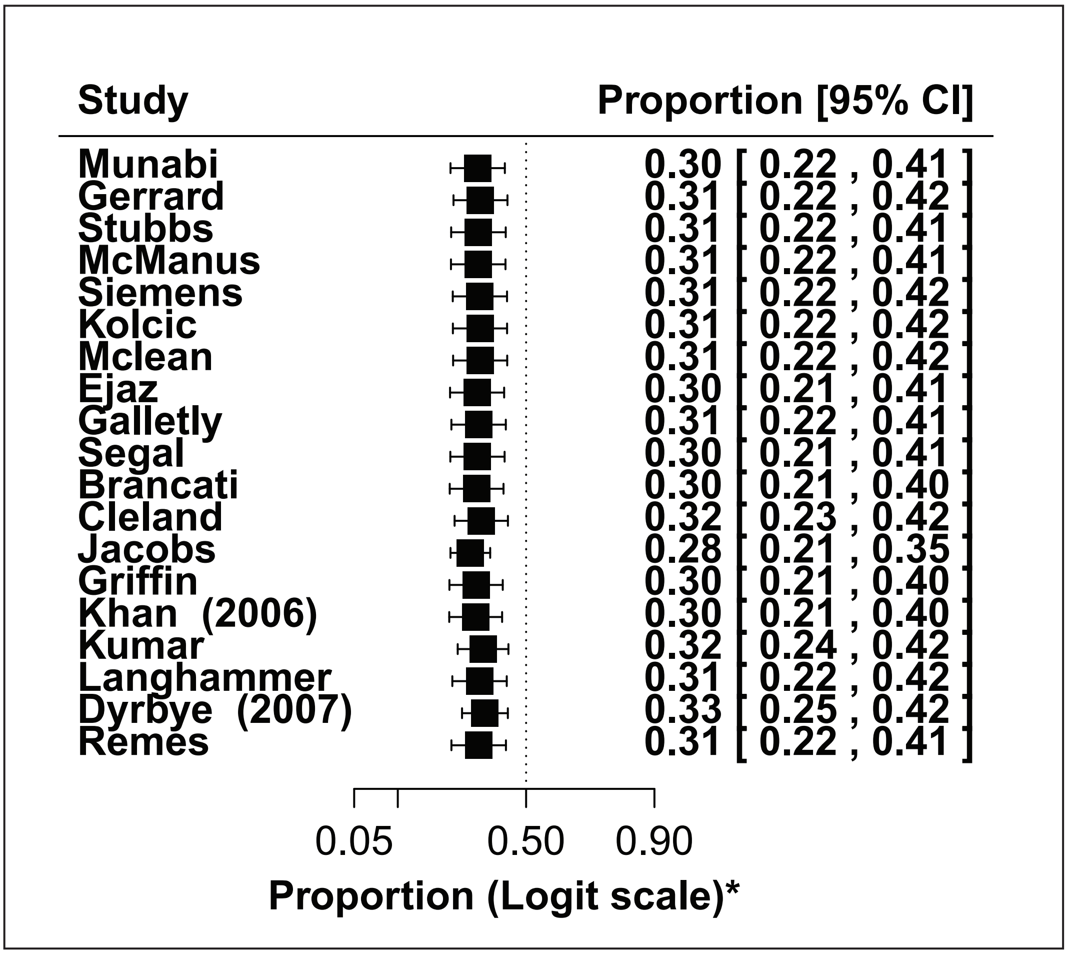

II.1.a. Positive effect of research experience on interest in a research career :

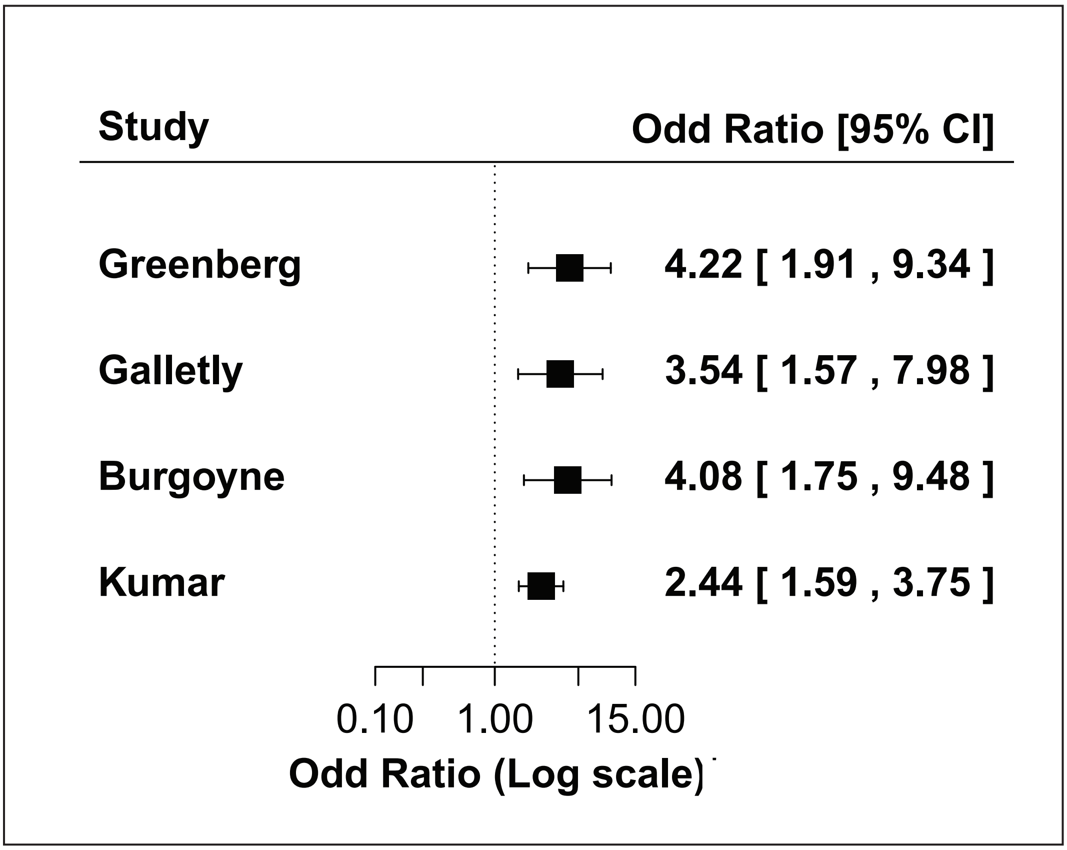

II.1.b. Positive effect of research experience on research attitudes or motivation :

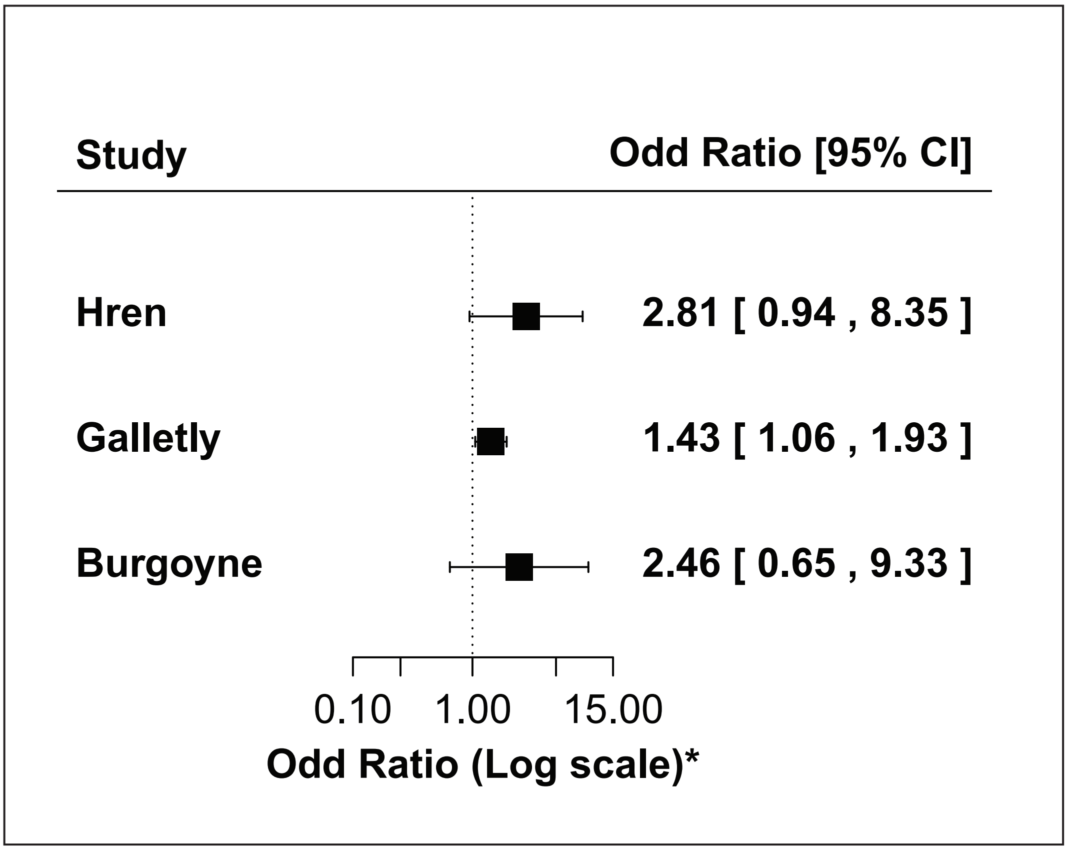

II.2.b. Academic success is associated with research involvement :

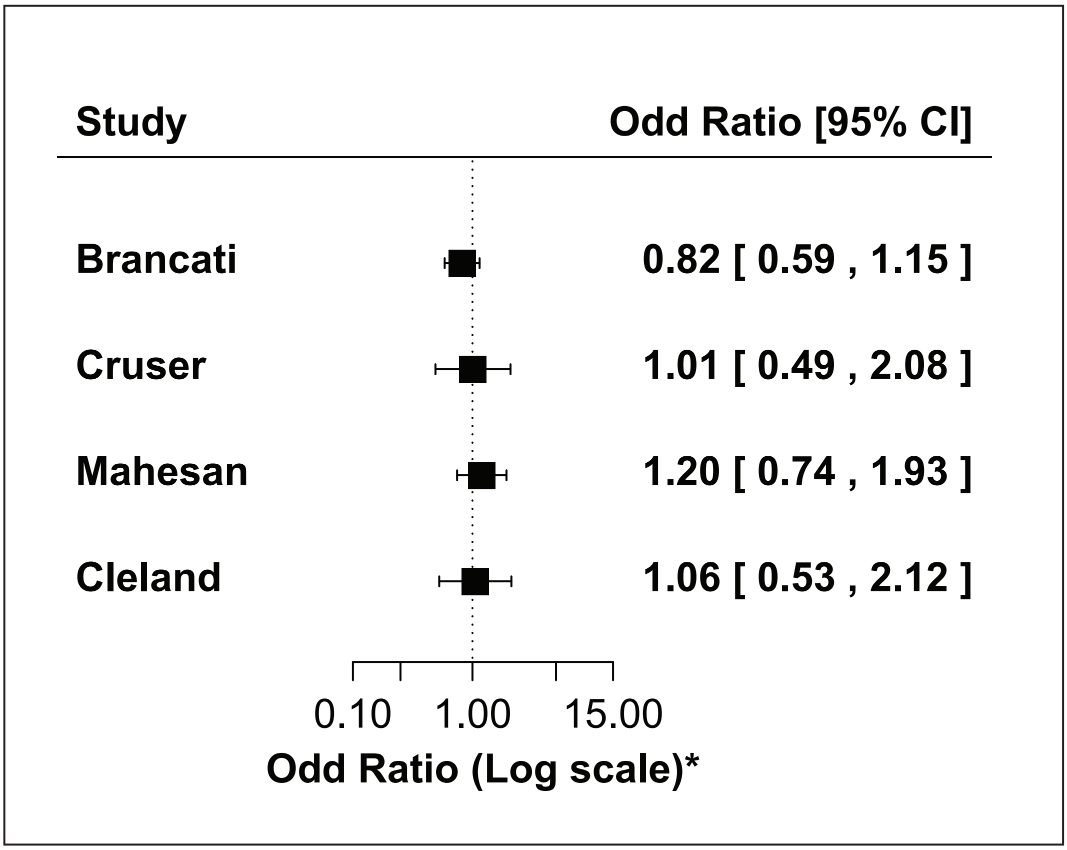

III.1.a: Medical student research results in a publication (All) :

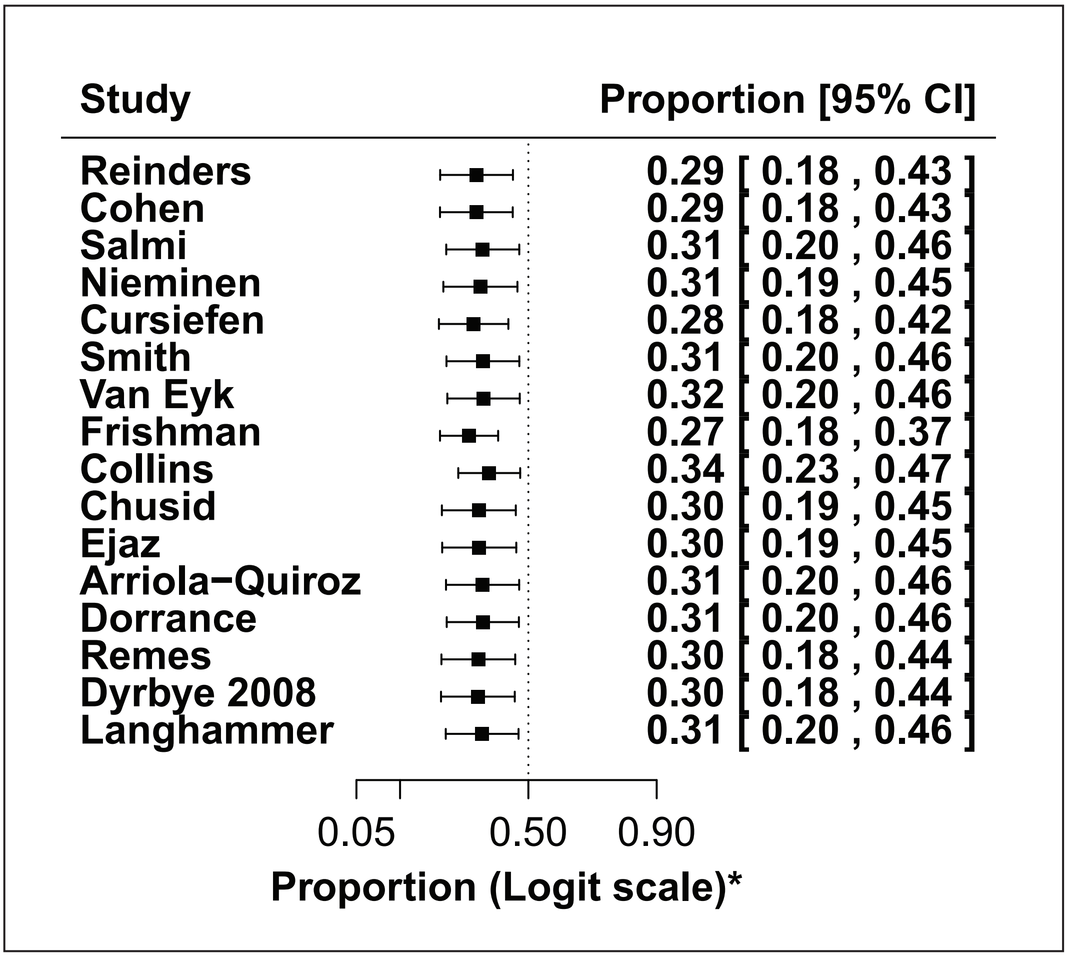

III.1.a: Medical student research results in a high quality publication :

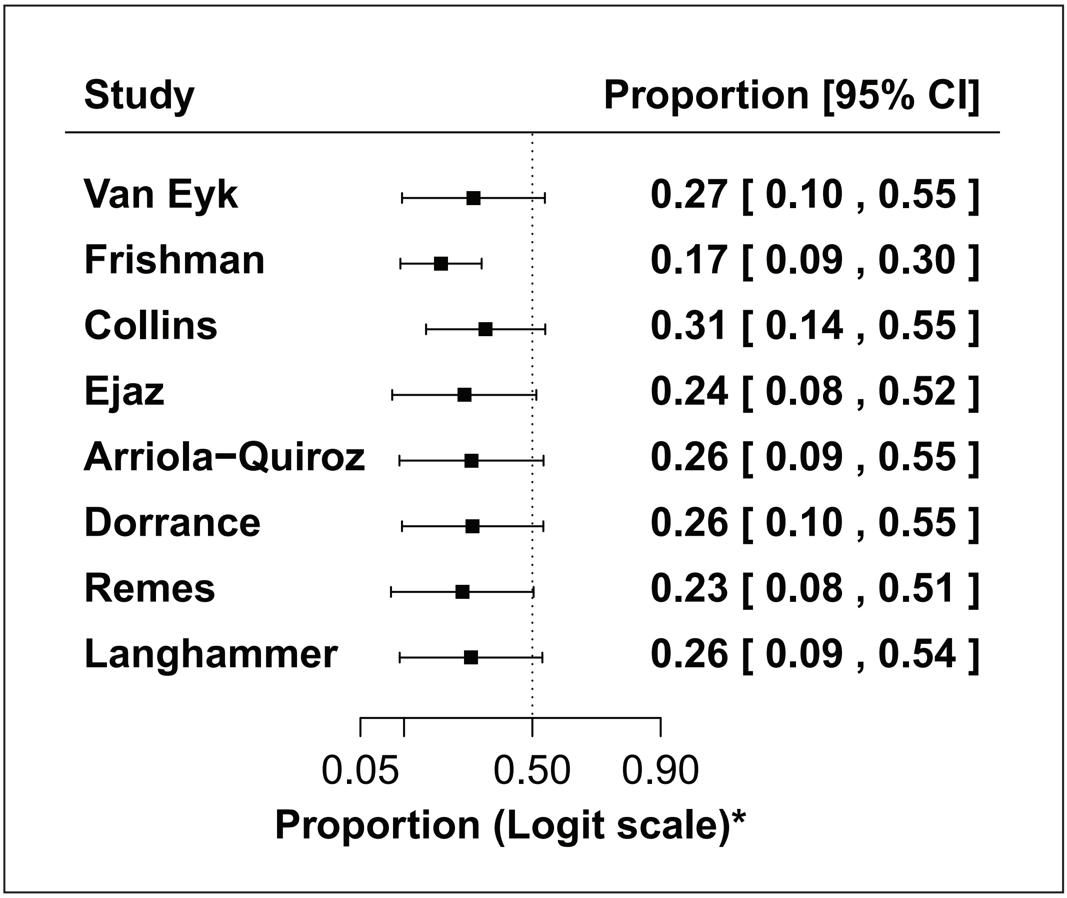

III.1.b: Medical student research results in a first-authored publication (All) :

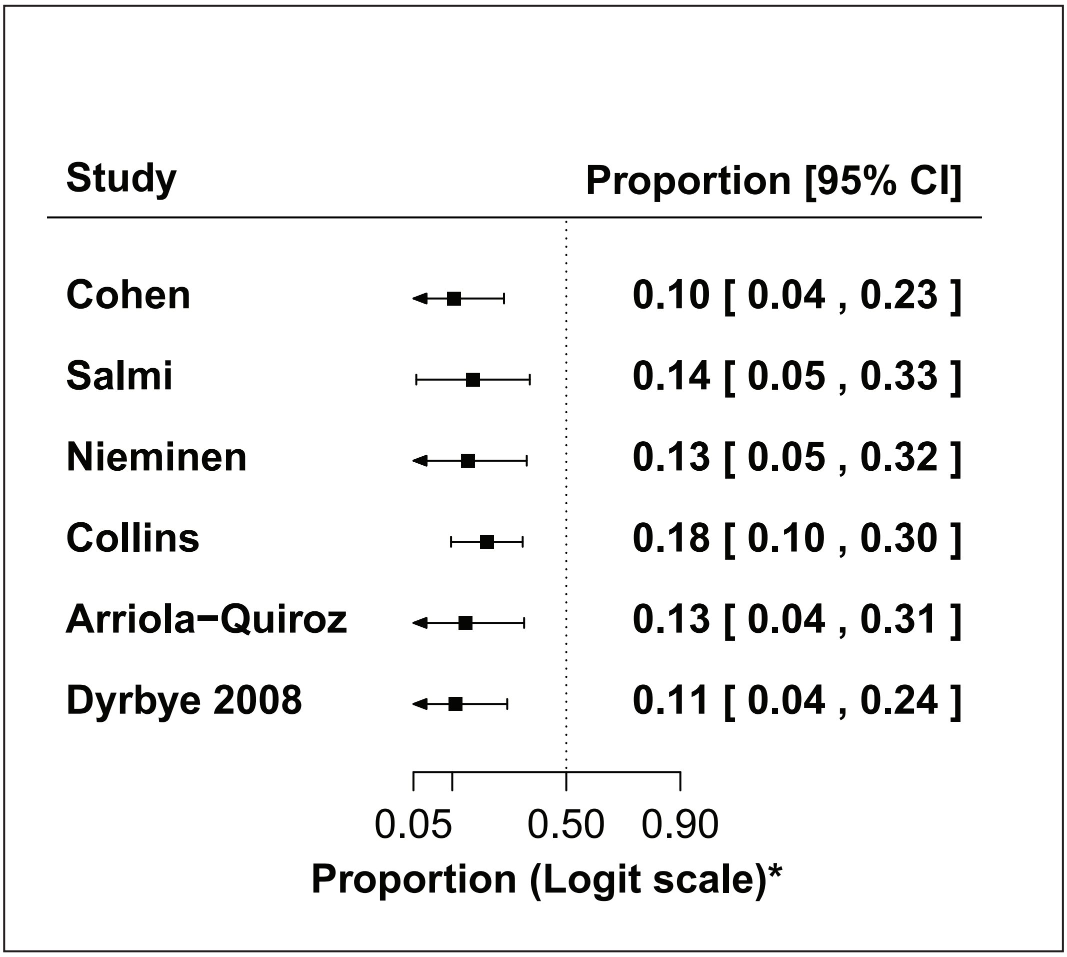

III.1.b: Medical student research results in a first-authored high quality publication :

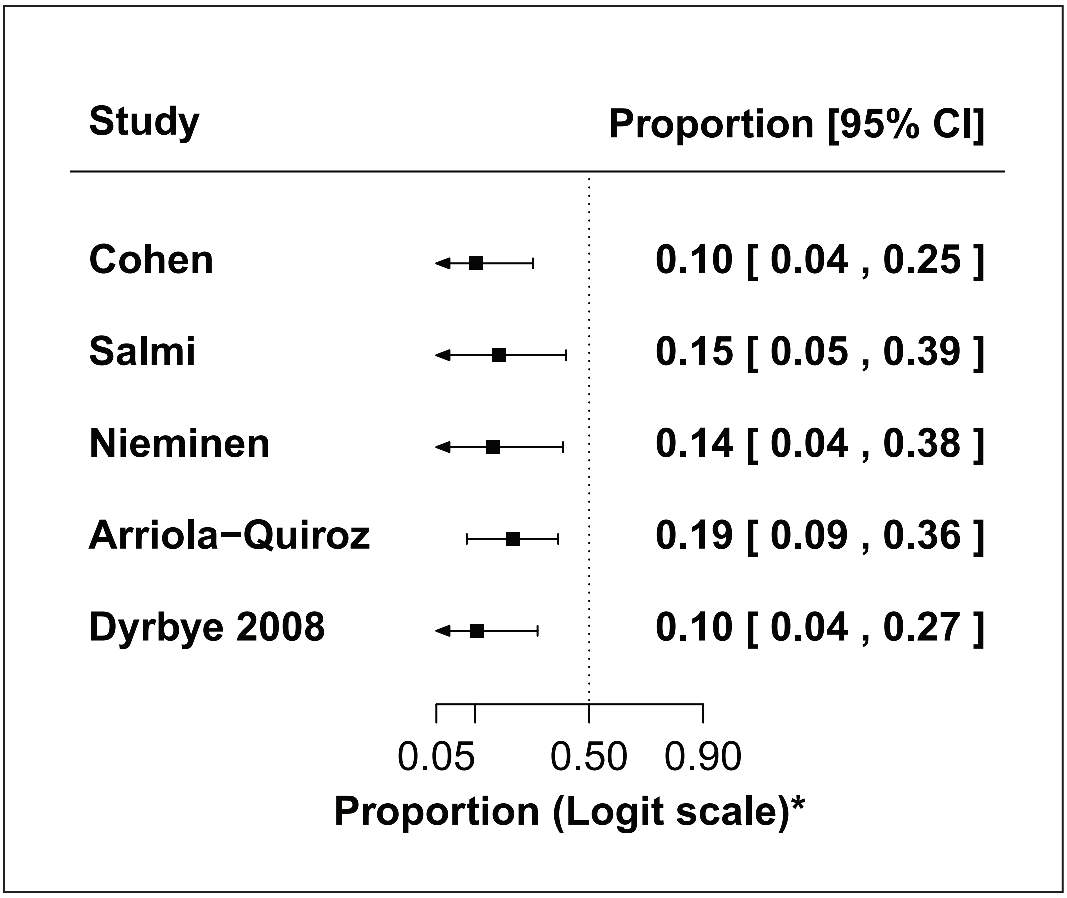

III.2: Associated later involvement in research projects (all):

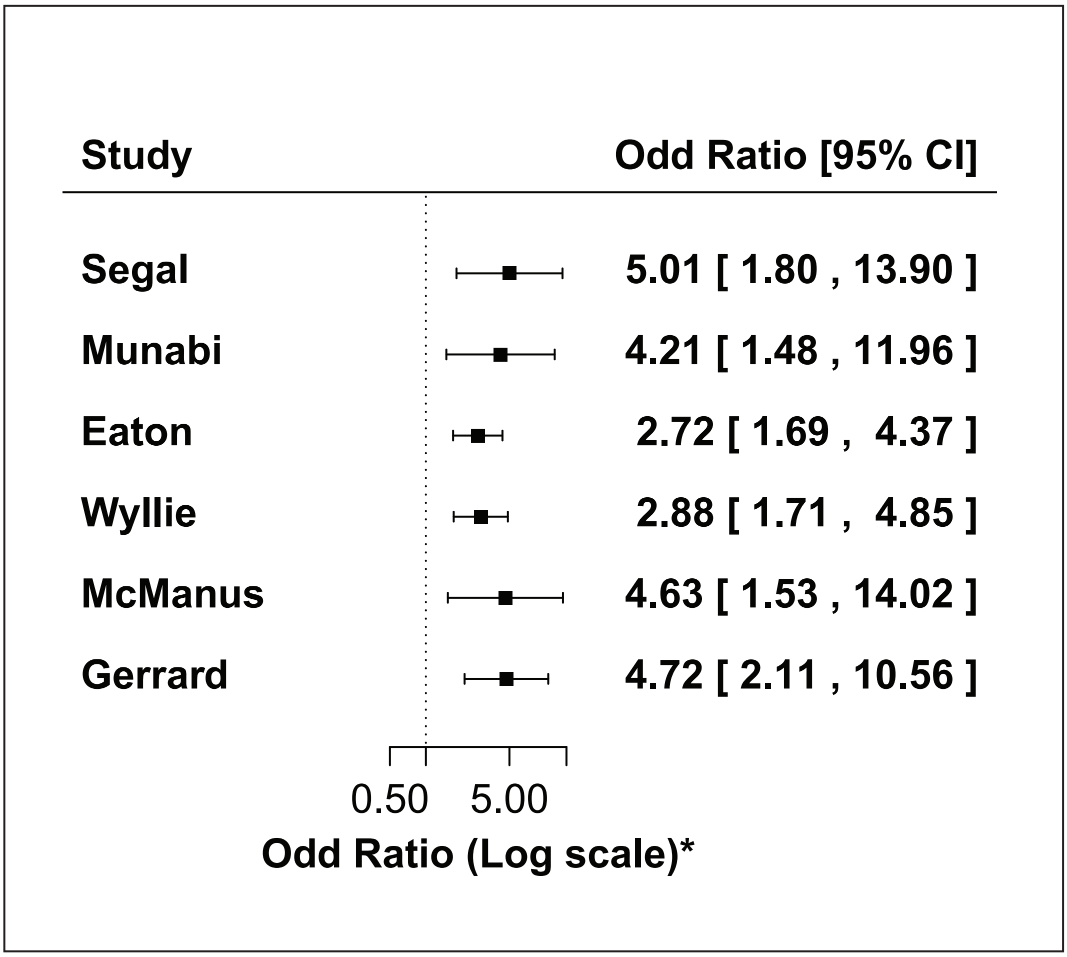

III.2.a: Associated choice of an academic career :

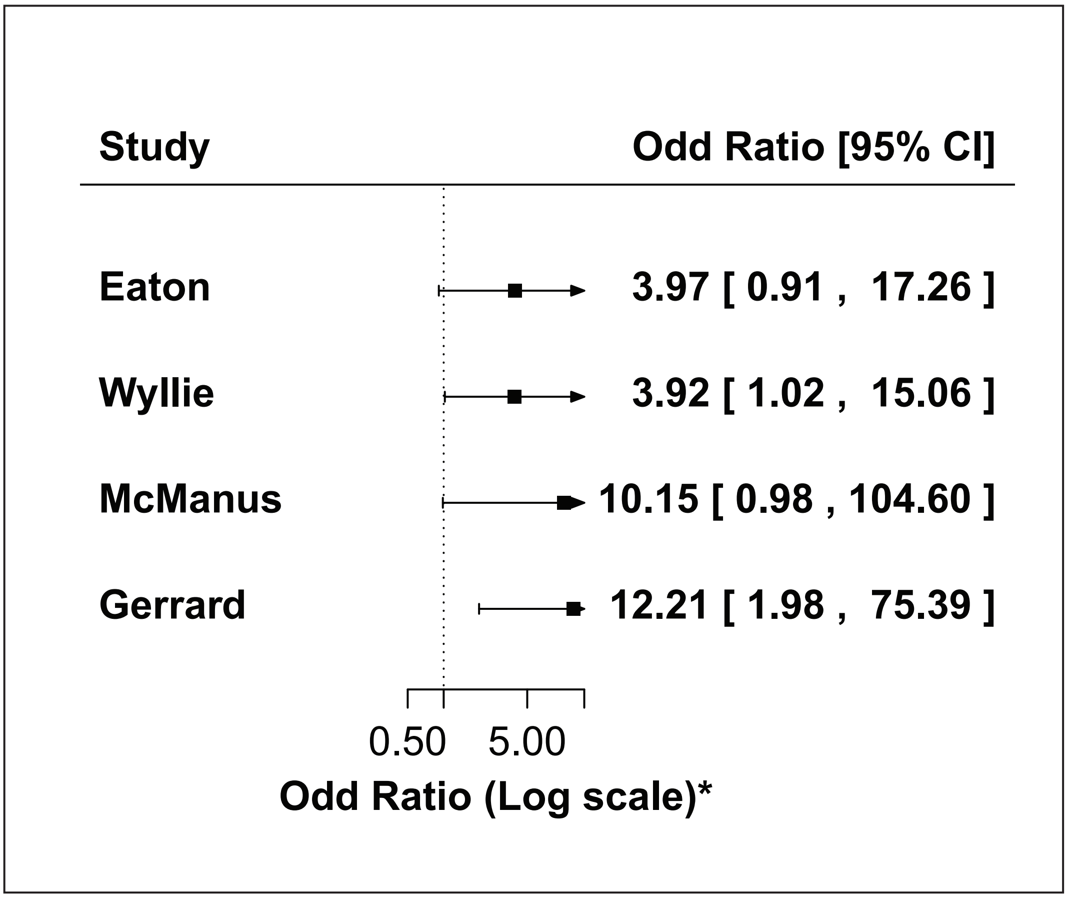

III.3: Associated higher odds of having at least one publication (long-term):

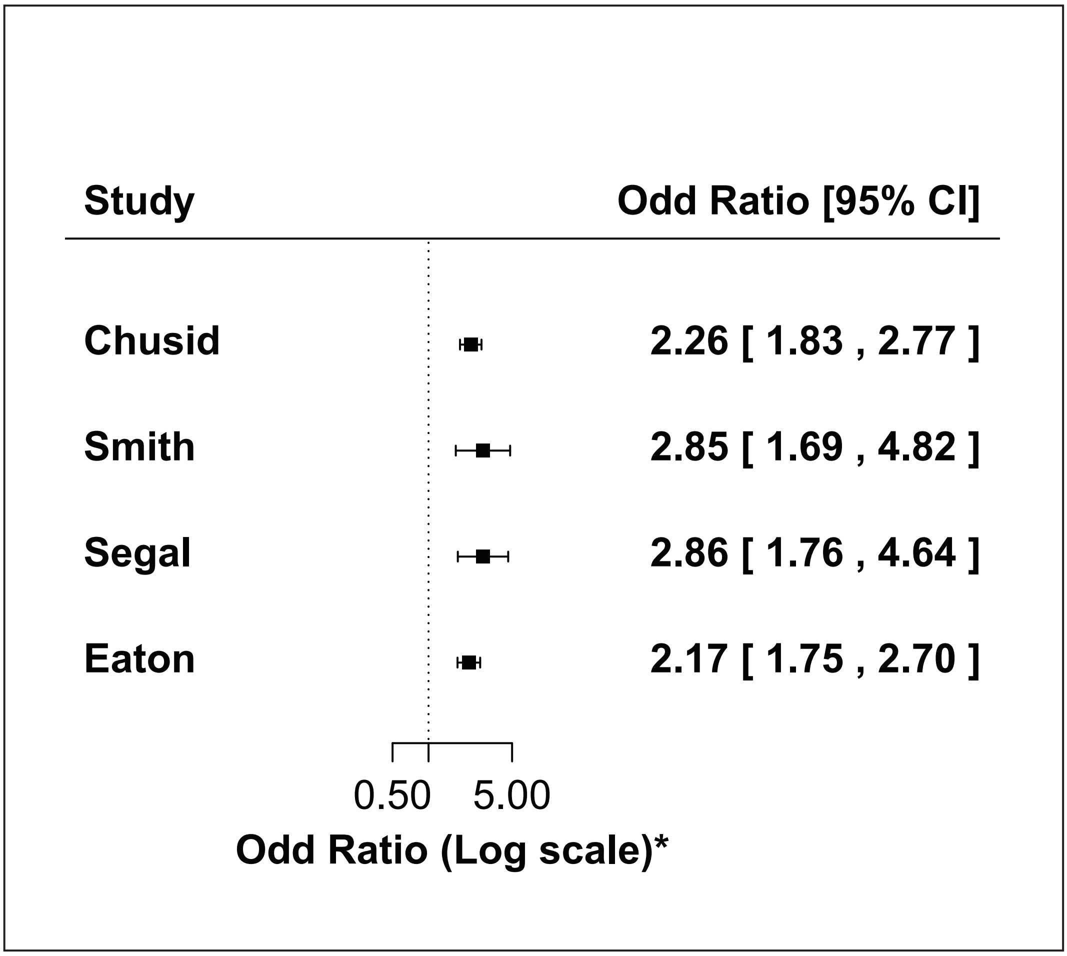

III.4: Associated higher interest in a related specialty :

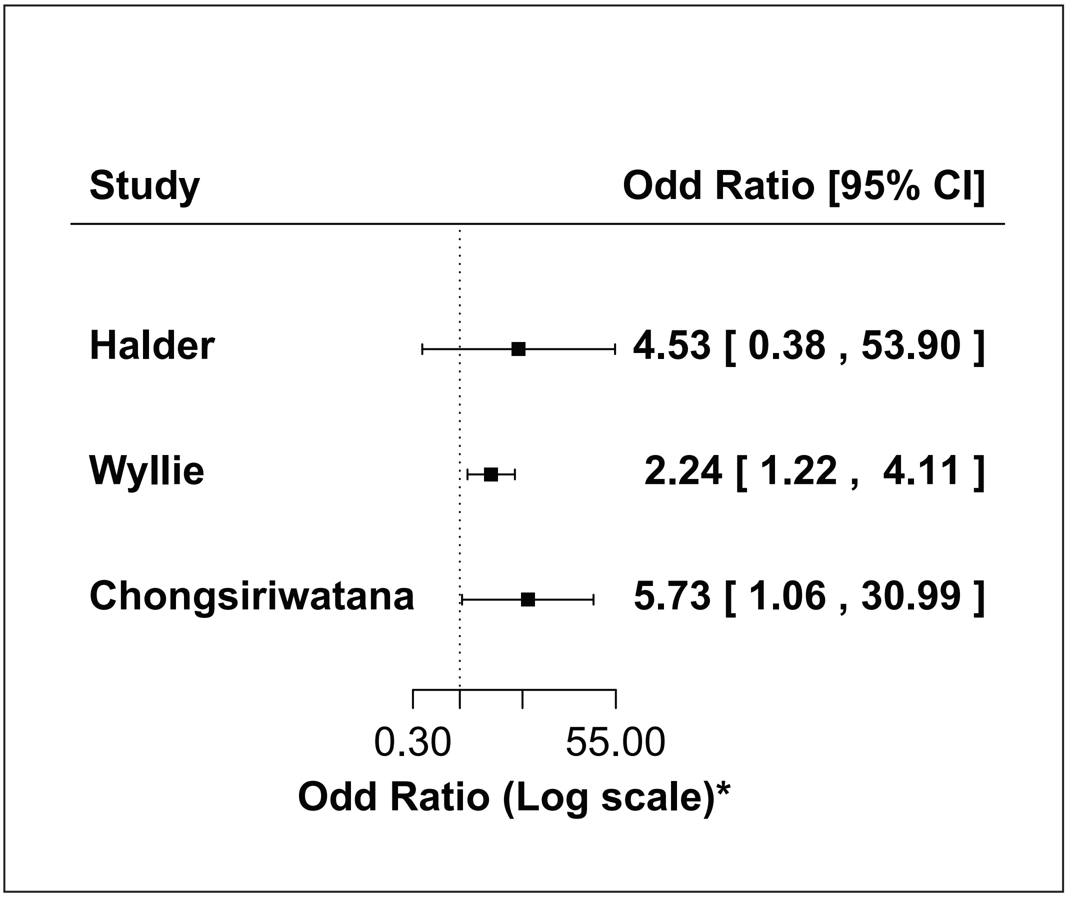

IV.1: Effect of research time-off on academic performance (final year + controlled) :

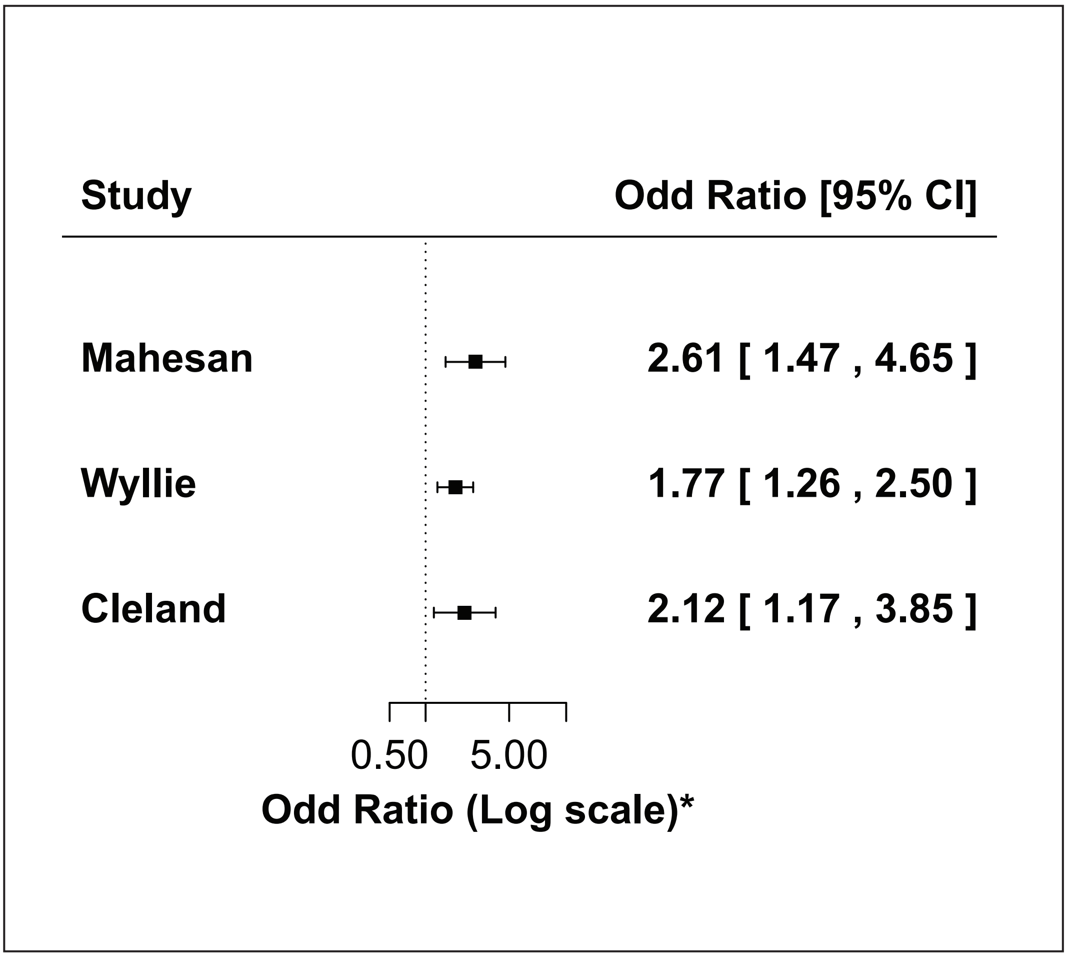

IV.2.a.: Gender differences in publication of research (males are "treated" group) :

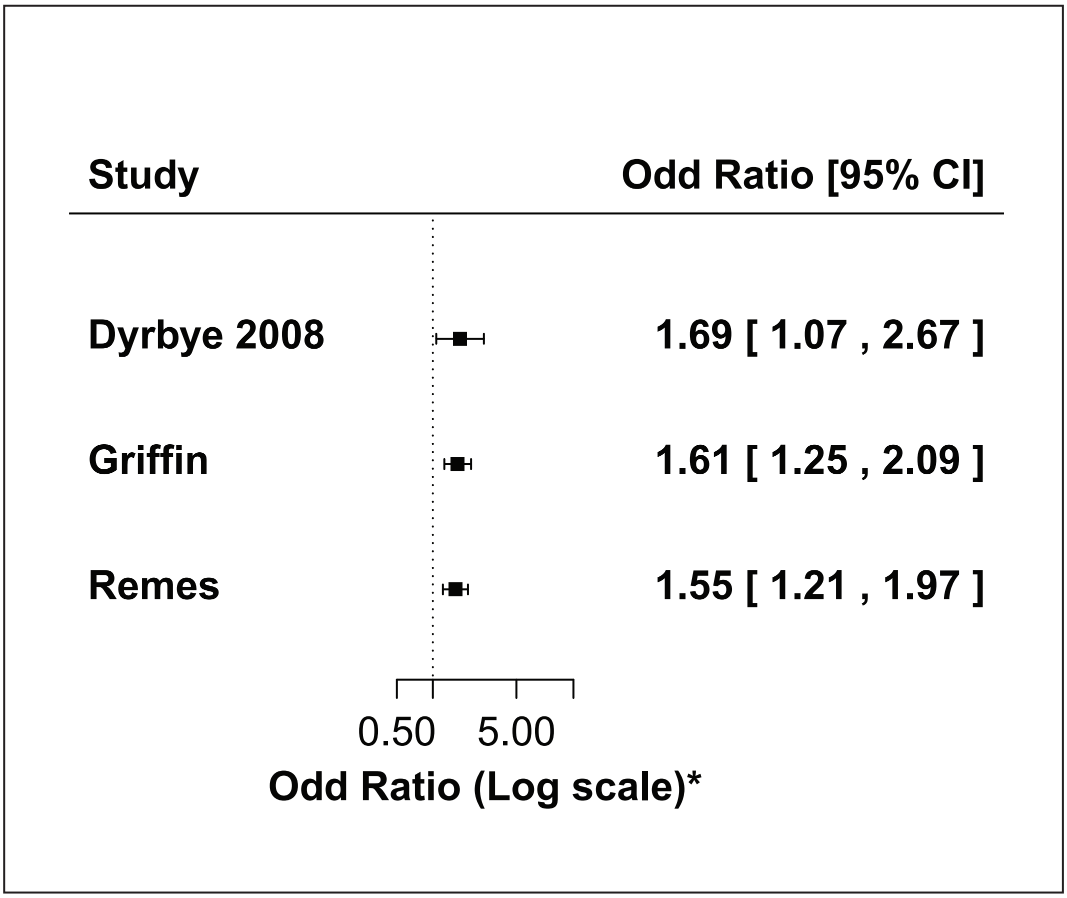

IV.2.b.: Gender differences in interest in a research career (males are “treated” group) :

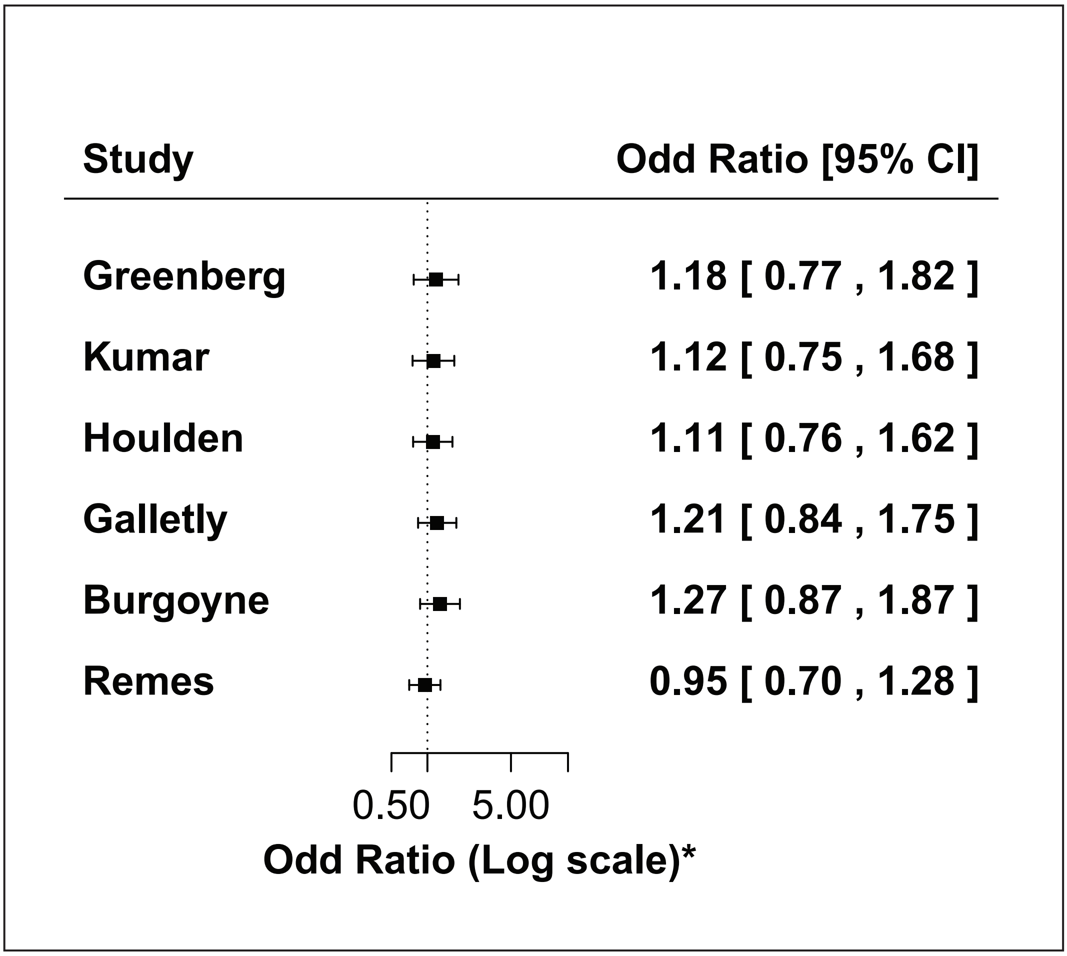

IV.2.c.: Gender differences in research involvement (males are “treated” group) :

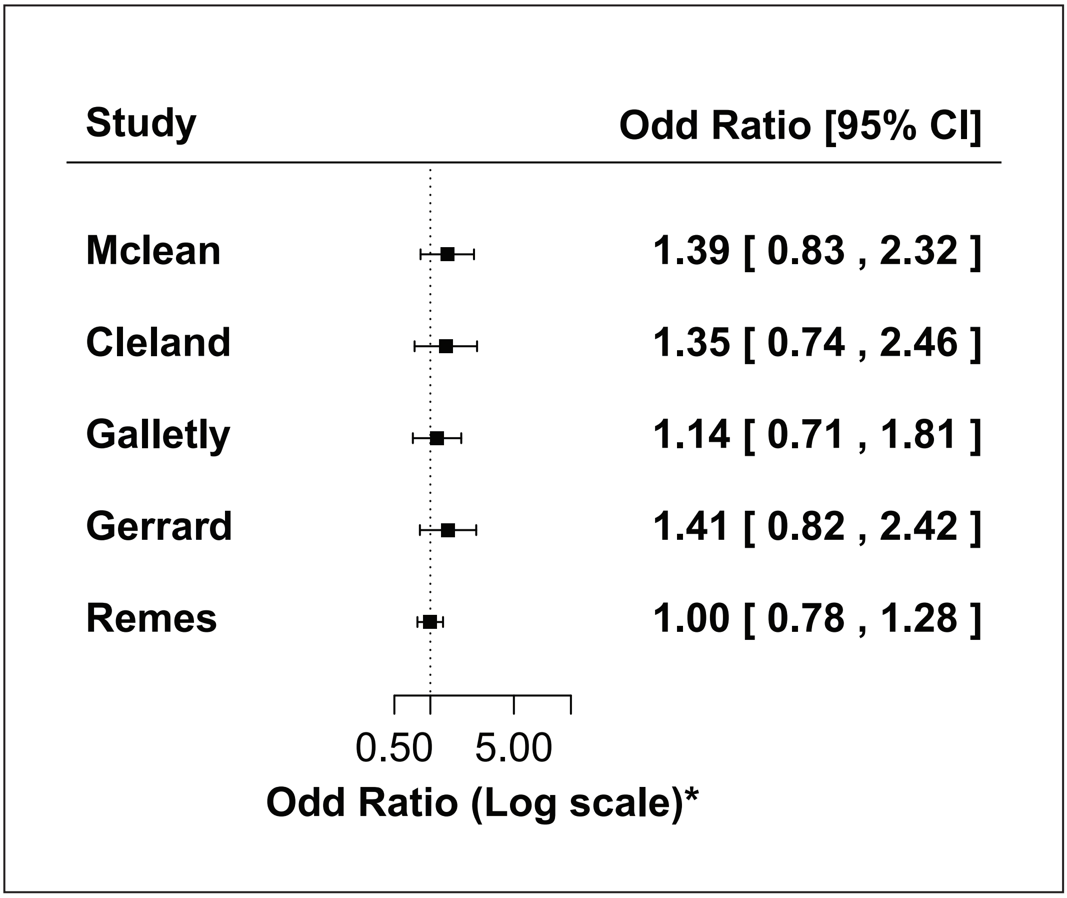

IV.2.d.: Gender differences in research attitudes (males are "treated" group) :

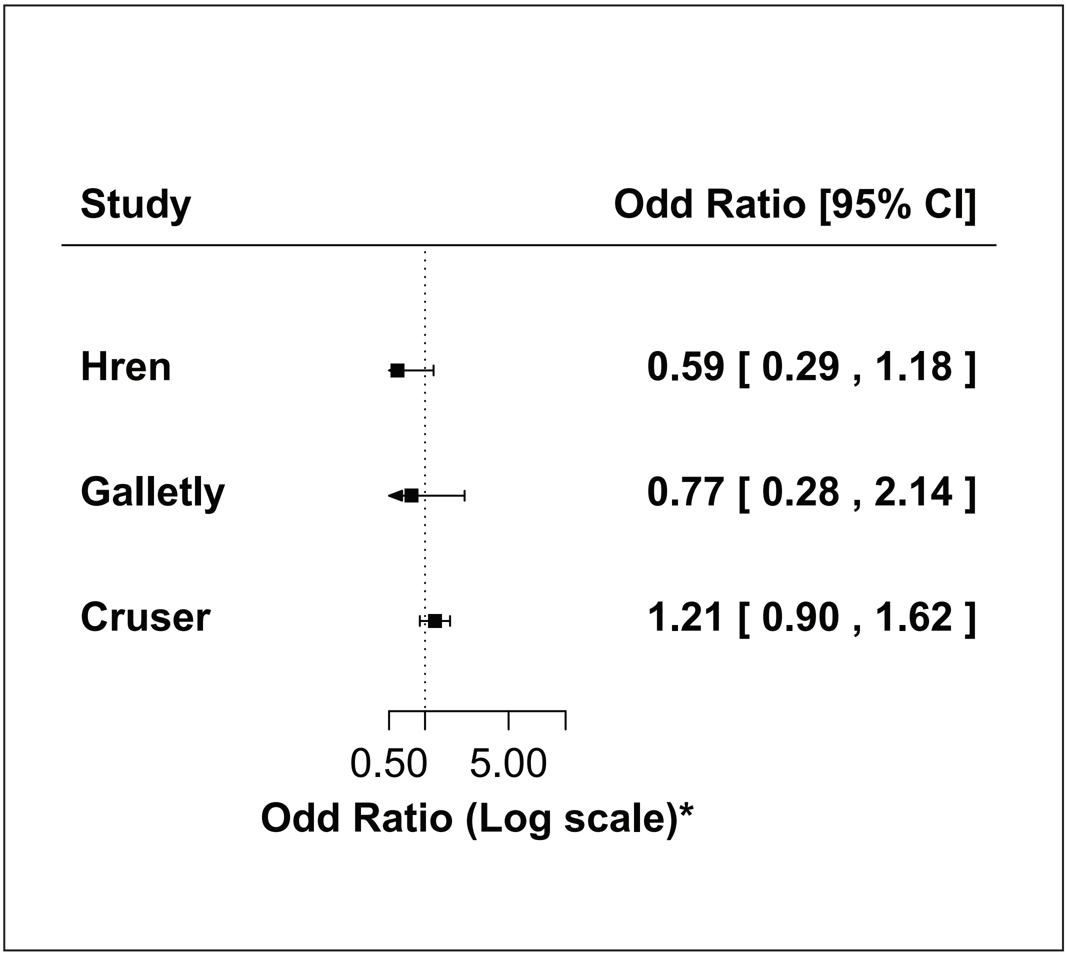

IV.2.e.: Gender differences in research interest (males are "treated" group) :

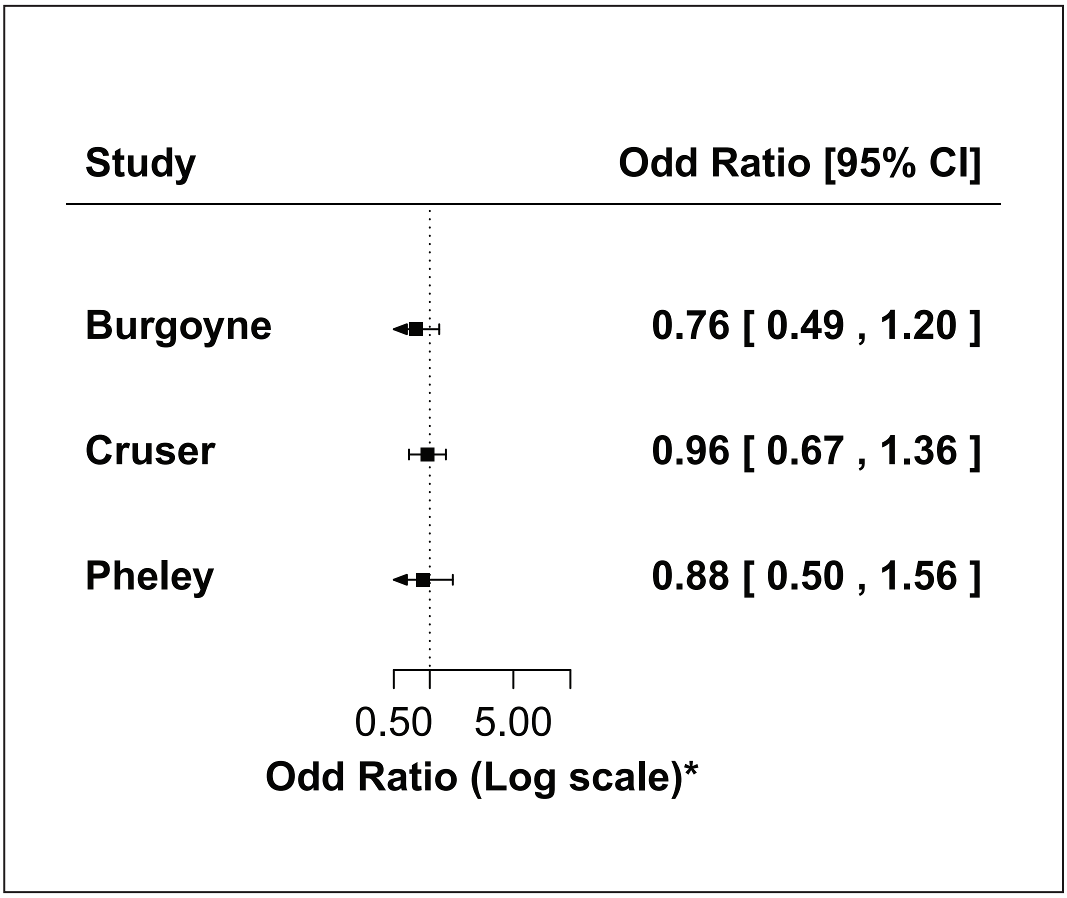

Supplement: S4 File — (PDF) [file pone.0127470.s004.pdf]
