# Supplementary material for: Medical Student Research: An Integrated Mixed-Methods Systematic Review and Meta-Analysis
Source: PLoS One. 2015 Jun 18;10(6):e0127470. doi: 10.1371/journal.pone.0127470 (PMC4472353; doi:10.1371/journal.pone.0127470)
Supplement: S5 File — (PDF) [file pone.0127470.s005.pdf]

SUPPLEMENTARY TABLES

Medical Student Research: An Integrated Mixed-Methods Systematic Review and Metaanalysis

Mohamed Amgad[1 2], Marco Man Kin Tsui[2], Sarah J. Liptrott[3] and Emad Shash[4 5]

1. *Faculty of Medicine, Cairo University, Cairo, Egypt*
2. *Okinawa Institute of Science and Technology Graduate University, Japan*
3. *European Institute of Oncology (IEO), Milano, Italy*
4. *National Cancer Institute, Cairo University, Egypt*
5. *European Organization For Research & Treatment of Cancer (EORTC) Headquarters, Brussels, Belgium*

Study	Type	Design	Institutions	N	Population	Outcome measure	Outcome
Burgoyne	X	Q	S	317	Medical students; University College Cork, Ireland	Were unaware of research at their own university.	257 (81.1%)
Griffin	X	Q	M	515	Students; seven medical schools in UK.	Were not encouraged by superiors to perform research.	319 (61.9%)
Dewey	X	Q	S	302	Students; University Hospital Charite	Poor supervision	166 (55.0%)
De Olivera	X	Q	M	978	Students; 13 medical schools (nine public schools and four private ones); Six Brazilian states.	Cited lack of institutional incentive as the main reason for lack of involvement in research.	323 (33.0%)
Munabi	X	Q	S	170	Undergraduates; Makerere University School of Medicine.	Lack of collaboration	118 (69.4%)
				161		Lack of funding	98 (60.9%)
				163		Lack of guidance	80 (49.1%)
Sanchez	X	Q	M	601	Students; Three national student conferences in the US	No sufficient guidance to develop publishable research product	152 (25.3%)
Galletly	X	Q	S	155	Final year students; University of Adelaide.	Lack of enough academic role models	109 (70.3%)
						Inadequate training	96 (61.9%)
Yamazaki	X	Q	S	33	Students; Juntendo University School of Medicine, Japan	Poor mentorship	1 (3.0%)
						Lack of academic role models	5 (15.2%)
						Lack of training	2 (6.1%)
						Lack of opportunities	9 (27.3%)
Remes	X	Q	S	91	Students; University of Helsinki	Insufficient supervision or poor mentorship	37 (40.7%)
Siemens	X	Q	M	327	Second- and Fourth- year medical students; Three Canadian Medical Schools.	Inadequate training	35 (10.7%)
						Inadequate mentorship	141 (43.1%)
						Lack of opportunities	64 (19.6%)

Supplementary Table 1: Some of the drawbacks mentioned by medical students. ***Abbreviations used:*** **X,** Cross-sectional; **Q,** Questionnaire; **S,** Single; **M,** Multiple.

Study	Type	Design	Control group	Institutions	Other Limitations	Overall quality	N	Population	Outcome measure	Outcome
**Jacobs**	R	Q	N	S		Low	73	Graduating class; Stanford University in 1991.	Various skills	73-94%
**Frishman**	R	Q	N	S		Low	69	Students; Albert Einstein College of Medicine	Data collection skills	63 (91%)
									Literature search	63 (91%)
									Research techniques	66 (95%)
**Zorzi**	R	Q	N	S	Methodology inadequately explained; Low response rate	Low	22	Participants; Rural Summer Studentship Program; University of Western Ontario; 1999-2003.	Knowledge	3.4-6.0 (Likert scale, 7 is max)
**Zier**	R	Q	N	S	Compulsory questionnaire (risk of careless reporting)	Low	111	Students who got funding for research; Mount Sinai Medical School *	Knowledge	100 (90%)
**Collins ***	X	Q	N	S	Methodology inadequately explained	Low	130	Medical students, University of Melbourne	Research skills	4.0 (Likert scale, 5 is max)
									Professional development	3.9 (Likert scale, 5 is max)
									Personal skills	3.7 (Likert scale, 5 is max)
**Wagner**	R	Q	***P***	S	Very small sample size	Low	5	Participants; Dermatology electives, University of Texas	Research skills	1.77-3.72 (Likert scale, 5 is max); P=0.05
**Riley**	I/Pro	Q	N	S		Medium	912	Students; University of Edinburgh; Effect of Student Selected Components (SCC's)	Knowledge	840 (92.1%)
**Burgoyne**	R	Q	Y†	S		High	316	Medical students; University College Cork, Ireland **	General skills Vs. exposure to research	OR=1.23 (0.61-2.46) ‡
							314		Research skills Vs. exposure to research	OR=0.9(0.51-1.58) ‡
**Stubbs ***	R	Q	N	M	Low response rate	Low	253	Students who chose to intercalate; Bristol and Sheffield Medical Schools, UK	Gain of skills useful for future career	230 (90.9%)
**Eaton ***	R	Q	N	S		Low	90	BMedSci (iBSc) graduates; University of Queensland, Australia	Gain of understanding of research methods	71 (78.9%)
**Galletly**	R	Q	N	S		Low	155	Final year students; University of Adelaide.	Knowledge	116 (75%)

Supplementary Table 2: Positive effect of research experience on medical students' knowledge or skills. * Studies describing the effect of an intercalated Bachelor of Science (iBSc)**; **** Research experience not necessarily during medical school (not specified) ;† Data obtained directly from authors and dichotomized (results are adjusted for age, sex and having a previous degree prior to matriculation)**; ‡** Results are not significant (we could not find any correlation). ***Abbreviations used:* X,** Cross-sectional; **R,** Retrospective; **I/Pro,** Interventional/Prospective; **Q,** Questionnaire; **Y,** Yes; **N**, No; **P,** Paired outcomes; **S,** Single; **M,** Multiple.

Study	Type	Design	Control group	Inst.	Other limitations	Overall quality	N	Population	Outcome measure	Outcome
**Kim**	X	Q	Y	M		Medium	1388	Students; Six Korean Medical schools	*Less* interest in clinical medicine among students planning non-clinical careers.	P<0.01
**Sanchez**	R	Q	Y	M	Low response rate; Base population is attendees of medical student conferences (interested in research almost by definition)	Medium	601	Students; Three national student conferences in the US	Students interested in primary care specialties significantly *less* interested in academic medicine career.	OR=0.46(0.32-0.64); P<0.0001
**Lloyd**	X/R	Q	Y	S	Low response rate; Base population is medical graduates (not students).	Low	433	Graduates; Penn State College of Medicine; 1985-95	Full time clinical practice *not* associated with an academic health center Vs. Involvement in clinical research	OR=0.13(0.08-0.20); P<0.0001
**Yamazaki**	X	Q	N	S	Low response rate	Low	33	Students; Juntendo University School of Medicine, Japan	Desire to be clinicians cited as an obstacle to entering basic science	22 (66.7%)

Supplementary Table 3: Disparity between interest in clinical practice and interest in a research career. ***Abbreviations used:*** **X,** Cross-sectional; **R,** Retrospective; **Q,** Questionnaire; **Y,** Yes; **N**, No; **Inst.**, Number of institutions; **S,** Single; **M,** Multiple.

Study	Type	Design	Control group	Control for confounding	Institutions	Lim.	Overall quality	N	Population	Outcome measure	Outcome
**Galletly**	X	Q	Y	Y *	S		Medium	105	Final year students; University of Adelaide.	Students interested in formal teaching have higher interest in performing research during their career	OR=3.62(1.41-9.33)
**Yamazaki ****	X	Q	Y	N	S	Rp.	Medium	269	Students; Juntendo University School of Medicine, Japan	Interest in basic sciences	OR=16.1(7.83-33.3)
**Kim**	X	Q	N	N	M		Low	42	Students; Six Korean Medical schools	Interest in research among those interested in basic sciences	13 (31%)

Supplementary Table 4 : Interest in academia (basic medical sciences or formal teaching) is related to interest in research. * Adjusted for age, sex and research involvement (data obtained directly from authors and dichotomized). ****** Data regrouped from original table presented***. Abbreviations used:*** **X,** Cross-sectional; **Q,** Questionnaire; **Y,** Yes; **N**, No; **S,** Single; **M,** Multiple; **Lim,** Other limitations; **Rp,** low response rate (<60%).
